# Distribution of a Community of Mammals in Relation to Roads and Other Human Disturbances in Gabon, Central Africa

**DOI:** 10.1111/cobi.12017

**Published:** 2013-02-14

**Authors:** Hadrien Vanthomme, Joseph Kolowski, Lisa Korte, Alfonso Alonso

**Affiliations:** Center for Conservation, Education and Sustainability, Smithsonian Conservation Biology InstituteNational Zoological Park, P.O. Box 37012, MRC 0705, Washington, D.C., 20013-7012, U.S.A

**Keywords:** abundance index, camera trapping, generalized models, hunting, mosaic landscape, oil extraction, road mitigation, cámaras trampa, caza, extracción de petróleo, índice de abundancia, mitigación de impacto de carreteras, modelos generalizados, paisaje de mosaico

## Abstract

**Abstract:**

We present the first community-level study of the associations of both roads and other human disturbances with the distribution of mammals in Gabon (central Africa). Our study site was in an oil concession within a littoral mosaic landscape. We conducted surveys along 199 line transects and installed camera traps on 99 of these transects to document mammal presence and abundance. We used generalized linear mixed-effect models to document associations between variables related to the ecosystem (land cover, topography, and hydrology), roads (coating, width of rights of way, condition, type of vehicle used on the road, traffic level, affiliation of users, and general type of road), and other human disturbances (urbanization, agriculture, hunting, logging, gathering, and industrial activities) and the abundance or presence of 17 species or groups of mammals including elephant (*Loxodonta cyclotis*), buffalo (*Syncerus caffer*), sitatunga (*Tragelaphus spekei*), red river hog (*Potamochoerus porcus*), smaller ungulates, gorilla (*Gorilla gorilla*), chimpanzee (*Pan troglodytes*), side-striped jackal (*Canis adustus*), carnivores, monkeys, and large rodents. Some types of roads and other human disturbances were negatively associated with the abundance or presence of elephants, buffalos, gorillas, sitatungas, some monkeys, and duikers. The pattern of associations of mammals with roads and other human disturbances was diverse and included positive associations with road presence (red river hog, some monkeys, and duikers), agriculture (sitatunga, small carnivores, and large rodents) and industrial activities (sitatunga, red river hog, red duikers, and side-striped jackal). Our results suggest that the community of mammals we studied was mostly affected by hunting, agriculture, and urbanization, which are facilitated by road presence. We recommend increased regulation of agriculture, hunting, and road building in the area.

Distribución de una Comunidad de Mamíferos en Relación a Carreteras y Otras Perturbaciones Humanas en Gabón, Africa Central

**Resumen:**

Presentamos el primer estudio a nivel de comunidad de la relación entre carreteras y otras perturbaciones humanas con la distribución de mamíferos en Gabón (África central). Nuestro sitio de estudio está dentro de una concesión petrolera en un paisaje litoral heterogéneo. Realizamos muestreos a lo largo de 199 transectos lineales e instalamos cámaras trampa en 99 de ellos para documentar la presencia y abundancia de mamíferos. Utilizamos modelos lineales generalizados con efectos mixtos para documentar las asociaciones entre variables relacionadas con el ecosistema (cobertura de suelo, topografía e hidrología), carreteras (tipo de revestimiento, ancho de derecho de vía, condición, tipo de vehículos que utilizan la carretera, nivel de tráfico, afiliación de los usuarios y el tipo general de carretera) y otras perturbaciones humanas (urbanización, agricultura, caza, tala, recolecta y actividades industriales) y la abundancia o presencia de 17 especies o grupos de mamíferos incluyendo elefantes (Loxodonta cyclotis), búfalo (Syncerus caffer), sitatunga (Tragelaphus spekei), cerdo rojo de río (Potomochoerus porcus), ungulados pequeños, gorila (Gorilla gorilla), chimpancé (Pan troglodytes), chacal con rayas a los lados (Canis adustus), carnívoros, monos y roedores de talla grande. Ciertos tipos de carreteras y otras perturbaciones humanas estuvieron asociadas negativamente con la abundancia o presencia de elefantes, búfalos, gorilas, sitatungas, algunos monos y antílopes. Los patrones de asociación de mamíferos con carreteras y otras perturbaciones humanas fueron diversos e incluyen asociaciones positivas con la presencia de carreteras (cerdo rojo de río, algunos monos y antílopes), agricultura (sitatunga, carnívoros pequeños y roedores de talla grande) y actividades industriales (sitatunga, cerdo rojo de río, antílope rojo y chacal con rayas a los lados). Nuestros resultados sugieren que la comunidad de mamíferos que estudiamos fue afectada principalmente por la caza, agricultura y urbanización, que son facilitadas por la presencia de carreteras. Recomendamos una mayor regulación de la agricultura, caza y construcción de carreteras en el área.

## Introduction

The effects of roads on species diversity has received growing attention since the 1990s (Trombulak & Frissell 2000; Forman et al. 2003; Coffin 2007) and led to the emergence of the field of road ecology (Forman et al. 2003; Roedenbeck et al. 2007; van der Ree et al. 2011). Yet there remains a lack of studies at the community and ecosystem levels, a geographical bias toward North America and Europe (Fahrig & Rytwinski 2009; Robinson et al. 2010), an ecosystem bias toward tundra and boreal forests (Benítez-López et al. 2010), and a lack of studies that address potential confounding variables (variable related to 2 factors of interest that falsely obscures or accentuates the relation between those factors) (Meinert 1986; Roedenbeck et al. 2007; Fahrig & Rytwinski 2009).

The effects of roads on terrestrial animal communities appear to be highly variable and cumulative, to occur at various geographical and temporal scales, and to depend on the biology of the species considered, characteristics of the road network, and the ecosystem itself (Forman et al. 2003; Coffin 2007; Robinson et al. 2010). Across Africa expansion of roads follows expansion of logging and mining concessions. On this continent, roads facilitate access of hunters to otherwise unreachable areas and are thus considered a major threat to large and medium-sized mammals (Wilkie et al. 2000; Geist & Lambin 2002).

We investigated the associations of the presence of different types of roads with the distribution of a community of large and medium-sized mammals in a heterogeneous landscape of Gabon (central Africa). We explicitly accounted for potential confounding variables (ecosystem variables) and human-disturbance variables other than roads. We considered roads and other anthropogenic disturbances simultaneously to disentangle their respective associations with the distribution of the mammal community and to inform possible measures to mitigate effects of roads on mammals.

## Methods

### Study Site

**Figure 1:**
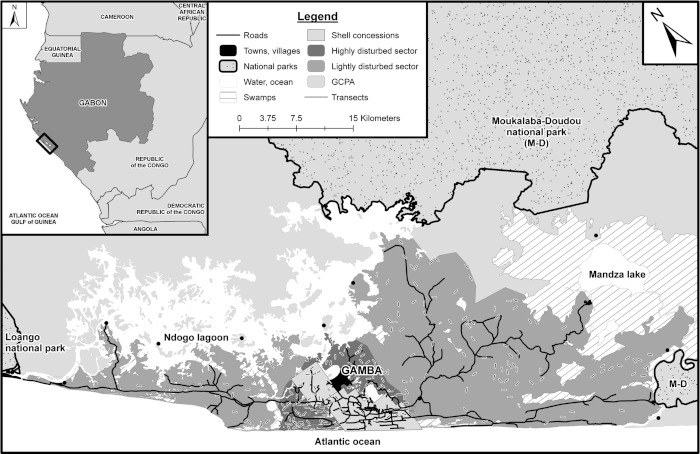
Location of the study area in relation to Gabon and the Gamba Complex of Protected Areas (GCPA).

The study site was in a mosaic of grassland, secondary forests, and wetlands of approximately 980 km^2^ along the Atlantic coast of Gabon (Fig. 1) and was centered on 2 onshore oil concessions (Gamba-Ivinga and Totou, managed by Shell Gabon) and the town of Gamba. These oil concessions are in the Gamba Complex of Protected Areas, which also includes Moukalaba-Doudou and Loango National Parks, logging and other oil-extraction concessions situated north of our study site. Annual rainfall averages approximately 2300 mm. There is a short dry season in January and a long dry season from late May to September. Oil extraction started in the Gamba area in the 1960s and has resulted in 3 primary current disturbances: oil-extraction activities sustained by a network of facilities (wells, pipelines, oil tanks, a port, administrative buildings, and lodging for employees); subsistence activities (agriculture, annual burning of savannas, gathering of nontimber forest products, hunting, fishing, wood harvesting) arising from the development of the Gamba town (≥9000 people) and satellite villages; and a network of mostly unpaved and freely accessible roads. Despite these disturbances, the area has a diverse community of large and medium-sized mammals, including African forest elephants (*Loxodonta cyclotis*), hippopotamuses (*Hippopotamus amphibius*), forest buffalos (*Syncerus caffer nanus*), sitatungas (*Tragelaphus spekei gratus*), smaller ungulates, leopards (*Panthera pardus*), western lowland gorillas (*Gorilla gorilla gorilla*), central African chimpanzees (*Pan troglodytes troglodytes*), carnivores, monkeys, and large rodents (Thibault & Blaney 2003; Boddicker 2006). Hunting is considered the main threat to these mammals in the area (Thibault & Blaney 2003) even though the area is protected and regulations are enforced (Gabon law forbids hunting some species and use of certain methods and specifies a hunting season).

### Sampling Scheme

In July 2010, we georeferenced all roads with a global positioning system (60CSx, Garmin, Olathe, Kansas) and described their 7 primary characteristics (Supporting Information): type of coating (tar, laterite, or sand), width of rights of way (16–20 m, 10–15 m, or <10 m), condition (good, degraded, or bad), type of vehicle used on the road (trucks, cars, or 4 × 4 vehicles), traffic level (≥11 vehicles/day, 2–10 vehicles/day, 1–7 vehicles/week, 1–4 vehicles/month, or <1 vehicle/month), affiliation of users (Shell staff members, general public, or both), and general type of road (major, production, restricted production, public, and vehicle track [Supporting Information]).

We used spatial data from Shell Gabon and local nongovernmental organizations and direct field verification to create current maps of land cover, hydrology, urbanization, and agriculture. We distinguished 2 sectors in our study area. The highly disturbed sector included the Gamba town and other surrounding villages (>60 persons/km^2^), most of the oil-operation facilities (lodging and administrative buildings, 0.7 wells/km^2^, 1.4 km of pipelines/km^2^), and a relatively high density of roads (1.0 km/km^2^). The lightly disturbed sector included fewer villages (<1 person/km^2^), fewer oil-operation facilities (no major building, <0.01 wells/km^2^, 0.02 km of pipelines/km^2^), and a lower road density (0.2 km of roads/km^2^) than the highly disturbed sector. We stratified our sampling by primary type of land cover (forest or savanna) and sector (highly or lightly disturbed). We excluded permanent swamps. Within each land-cover type and sector combination (4 strata), we randomly generated 50, 500-m line transects (our statistical unit), with centers at least 500 m apart, oriented parallel to the nearest road. After abandoning some transects due to inundation, we had 50 transects in savannas and 48 in forests in the highly disturbed sector and 50 in savannas and 51 in forests in the lightly disturbed sector (*n* = 199). We did all map editing and calculations of distance, areal extent, and density with ArcGIS 10.0 geographical information system (GIS) software (ESRI, Redlands, California) and with GME 0.5.5 β software (H.L. Beyer, Toronto, Ontario).

### Collection of Field Data

When physically clearing the understory on the transect lines (October 2010; <1 m wide), we recorded data on ecosystem characteristics (visibility of animals, land-cover type, understory type, canopy cover, level of inundation, topographic profile [Supporting Information]) (Laurance et al. 2008). We (a team of 3 people, including 2 highly experienced animal and track specialists and a field assistant) walked each transect between 600 and 1400, 15–30 days after the understory was cleared. We walked all transects slowly (about 1 km/h) and paused at least 1 minute every 100 m to listen for animal sounds.

We recorded all direct (e.g., visual or auditory observation) and indirect signs of mammal presence (e.g., tracks, feces) or human activity (e.g., footprints, rifle cartridge cases). For all signs, we recorded the species (or the closest taxon when equivocal). We recorded direct observations only if the group of animals or people was closer than 250 m to the transect (distance measured with a rangefinder [Rangemaster 1200CRF-Y, Leica, Allendale, New Jersey] for visual observations). We recorded indirect signs of animals only if the age of the sign (estimated on the basis of the team's experience) was <30 days. When age of the sign was equivocal, the sign was not recorded. We destroyed all signs after observation except for irremovable ones (e.g., nests of apes), which we marked with biodegradable flagging tape and only recorded once. We walked each transect twice in both wet (November 2010–January 2011) and dry (June–August 2011) seasons. There were 15–20 days between the 2 surveys within a season. We avoided sampling transects twice at the same time of day (reduce daily bias) and had different combinations of people in the team sample all transects (minimize observer bias among team combinations). Furthermore, H.V. was part of every team combination and confirmed species identification and age estimation for all signs.

We installed a camera trap (RC55 Rapidfire, Reconyx, Holmen, Wisconsin) near the midpoint (≤50 m) of each forest transect, where the transect crossed an animal trail and signs indicated recent animal presence. Animal trails were dense across the study area, and we found trails near the midpoint of all transects. Camera traps were approximately 50 cm aboveground and oriented to cover both the transect and the animal trail. Camera traps photographed animals walking on the transect and on the trail during the 15–20 days between the 2 surveys of each transect in each season. No cameras were installed on savanna transects.

### Model Variables

We used 3 types of variables to document species presence or abundance on transects (“distribution variables”): counts of signs on transects (number of observations of signs of individuals or groups [CST]); counts of trapping events (number of individuals photographed [CTE]); and presence or absence (1 if CST ≥ 1 or CTE ≥ 1; 0 otherwise [PA]). We created one distribution variable for each species (Table 1, rationale in Supporting Information) and calculated its value for each transect.

**Table 1:** Information used to calculate the distribution variable (response variable in models) for each of the 17 species or groups of mammals

Species or group	Type of	Land cover	Seasons	Monitoring data	Camera-trapping	Item
of species	variable^a^	considered^b^	grouped^c^	included^d^	data included	counted
Forest elephant *Loxodonta cyclotis*	PA	F+S	no	tr, f, o	no	groups
Buffalo *Syncerus caffer nanus*	CST	F+S	no	tr, f, rs, o	no	groups
Sitatunga *Tragelaphus spekei gratus*	CST	F+S	no	tr, f, rs, o	no	groups
Yellow-backed duiker *Cephalophus silvicultor*	PA	forest	yes	tr, f	yes	individuals
Blue duiker *Philantomba monticola*	CTE	forest	no	no	yes	individuals
Red duikers^e^	PA	forest	yes	tr, f	yes	individuals
Red river hog *Potamochoerus porcus*	CST	F+S	yes	tr, f, o, w	no	groups
Water chevrotain *Hyemoschus aquaticus*	PA	forest	yes	tr, f	yes	individuals
Side-striped jackal *Canis adustus lateralis*	PA	savanna	yes	tr, d, o	no	groups
Small terrestrial carnivores^f^	CST	savanna	yes	tr, f, o	no	groups
Western lowland gorilla *Gorilla gorilla gorilla*	PA	forest	yes	tr, fs, f, n	yes	individuals
Central African chimpanzee *Pan troglodytes troglodytes*	PA	forest	yes	n, tr, c, f, o	yes	individuals
Collared mangabey *Cercocebus torquatus*	PA	forest	no	c, o, tr, f	yes	groups
Spot-nosed monkey *Cercopithecus nictitans*	CST	forest	yes	c, o	no	groups
Common monkeys^g^	CST	forest	no	c, o, tr, f	no	groups
Brush-tailed porcupine *Atherurus africanus*	PA	forest	no	o	yes	individuals
Giant-pouched rat *Cricetomys emini*	PA	forest	no	no	yes	individuals

a Abbreviations: CST, counts of signs on transects or number of observations of signs of individuals or groups during monitoring of the transects; CTE, counts of trapping events or number of individuals photographed with camera traps; PA, presence or absence (1 if CST ≥ 1 or CTE ≥ 1; otherwise 0).

b Data from one or both land-cover types (F+S, forest and savanna).

c Seasons are wet (November 2010–January 2011) and dry (June–August 2011).

d Abbreviations: tr, tracks; f, feces; o, visual observation; c, calls; rs, resting sites; w, wallows; d, den; fs, feeding sites; n, nests.

e Cephalophus dorsalis, C. ogilbyi, C. nigrifrons, C. callipygus, and C. leucogaster.

f Civettictis civetta, Atilax paludinosus, Herpestes naso, H. sanguineus, Crossarchus platycephalus, and Bdeogale nigripes.

g Cercocebus torquatus, Cercopithecus nictitans, C. cephus, C. pogonias, and Miopithecus ogouensis.

We measured 13 covariates related to the ecosystem (“ecosystem variables”) and 8 variables related to human disturbances other than roads (“human-disturbance variables”) per transect. Except for the season variable, we calculated the values of ecosystem variables (Supporting Information) by aggregating the data collected during the physical clearing of the transects and with the GIS (3 variables for land-cover heterogeneity and distances to fresh water, the ocean, and permanent swamps). To calculate values of the industrial-activities variable, we counted during transect monitoring the number of roads, pipelines, quarries, and pieces of abandoned equipment (Supporting Information). To calculate values of the field-activities variable, we counted signs of hunting, fishing, gathering, and artisanal logging during transect monitoring. With the exception of sector, we calculated the other human-disturbance variables with the GIS (2 variables for distance to settlements and distances to agriculture, industrial facilities, and national parks).

We measured 3 general variables related to roads for each transect (“general road variables”) with the GIS: linear distance from the center of the transect to the nearest road (kilometers); road density in a 2.1-km radius around the transect line (kilometers per square kilometer); and road density in a 0.5-km radius around the transect line (kilometers per square kilometer). Then, for each of the general road variables, we calculated a family of 25 new variables (“road-characteristic variables”), one for each level of the 7 road characteristics (see Sampling Scheme). Thus, we measured 75 variables (3 families of 25 variables each), including, for example, distance to the nearest tar road, density of tar roads within 2.1 km, and density of tar roads within 0.5 km (Supporting Information).

### Data Analyses

For each species, we fitted a general linear model (GLM) with the distribution variable (CST, CTE, or PA) as the response variable. When the distribution variable was available for each season, we used a generalized linear mixed-effect model (GLMEM) to account for potential temporal pseudoreplication due to sampling of the transects in both seasons (Ramsey & Schafer 2002; Crawley 2007). We modeled count-distribution variables (CST and CTE) with a Poisson GLM or GLMEM (log-link and Poisson distribution of errors) and weighted CTE models by the inverse of the number of trapping days for each transect to account for unequal camera trapping effort in each transect. We modeled presence-absence distribution variables with a binomial GLM or GLMEM (logit-link and binomial distribution of errors) (Ramsey & Schafer 2002; Crawley 2007). We completed all statistical analyses in R (R Development Core Team 2011).

Our data analysis included 4 steps. In step 1, we attempted to control for variability in distribution that was unrelated to roads by fitting a baseline model for each species that included only the ecosystem and human-disturbance variables (Ramsey & Schafer 2002). We used Akaike's information criterion (AIC) (Akaike 1973) and a stepwise method (Ramsey & Schafer 2002; Crawley 2007) to simplify the model. For each species, we systematically removed one variable at a time from the model containing all ecosystem and human-disturbance variables. We then calculated the AIC for each simplified model and retained the model with the lowest AIC. The process was repeated until we obtained a null model. Due to the large number of variables, we did not include interaction effects or quadratic terms in the candidate models. Out of the approximately 200 models fitted for each species, we retained the one with lowest AIC (ΔAIC = 0) and all others with ΔAIC ≤ 2 (“step-1 candidate models”) for the next round of selection. For every step-1 candidate model, we calculated collinearity with the generalized variance-inflation factor (gVIF) (Fox & Monette 1992), overdispersion (for Poisson models) with parameter ϕ (Hilbe 2007), and spatial autocorrelation with a randomized Moran test (Sokal 1978; Fortin & Dale 2005). If we detected collinearity (variables with gVIF > 7), the model was eliminated from the pool of step-1 candidate models. If all step-1 candidate models had a variable with a gVIF > 7, we eliminated this variable and repeated the full procedure of fitting until collinearity was not detected in at least one step-1 candidate model (Zuur et al. 2010). For poisson models, we retained the step-1 candidate models for which no overdispersion was detected (ϕ ≍ 1). We ultimately retained one step-1 candidate model (“step-1 final model”) for each species on the basis of previous criteria and the following 3 qualities: no pattern was detected when plotting residuals against fitted values, number of human disturbance variables in the final model was maximized, and number of significant (*p* < 0.05) associations between covariates and response variable was maximized. We obtained a step-1 final model with a different assemblage of covariates for each species.

In step 2, we used the step-1 final model to assess the association of road variables with the distribution variable by adding one at a time each general road variable as a covariate. We selected one of the 3 resulting step-2 candidate models after checking collinearity (gVIF), overdispersion (ϕ for Poisson models), and spatial autocorrelation in the residuals (Moran's test) and on the basis of the following criterion: either there was a significant association between the distribution and general road variables or, if the latter association was not significant, the number of significant associations between the distribution and human-disturbance variables was maximized. We used this step-2 final model to document the associations between the general road and distribution variables. For each species, we tested the sensitivity of the models by adding the general road variable selected in step 2 to the 20 step-1 models with lowest AIC and compared the values of the estimated coefficients for the general road variable and their standard errors.

In step 3, we assessed the associations between road-characteristic variables and the distribution variable for each species by replacing the general road variable in the step-2 final model with the variables from the same family that described each level of a given characteristic of the road. For example, if the best final model included distance to the nearest road as the general road variable, we replaced it with the 3 variables giving the distances to the nearest roads of each type of coating (distances to the nearest tar, laterite, and sand roads). We then fitted 6 other models by replacing the general road variable in the step-2 final model with the variables accounting for each level of the 6 other characteristics of roads (in our example, distances to each level of rights of way, condition, type of vehicle, traffic, affiliations of users, and general type of roads). For the 7 models obtained, we documented whether associations between the 25 road characteristics variables and distribution variable were statistically significant.

In step 4, we documented the size and uncertainty of the effect of road and human-disturbance variables on distribution variables by calculating incident rate ratios (factor by which the count-distribution variable increases when the value of the covariate increases by one unit) for count-distribution variables (CST or CTE) and odds ratios (factor by which the expected probability of presence increases when the value of the covariate increases by one unit) for presence-absence distribution variables and their confidence intervals (Ramsey & Schafer 2002; Crawley 2007). Primarily for ease of interpretation, we grouped road–characteristic variables that were significantly associated with the distribution variable into 3 factors: main roads (tar or laterite coating, more than 15 m wide, good condition, driven by trucks or cars, used by staff of corporations, or categorized as major or production), secondary roads (sand coating, degraded or bad condition, driven by 4 × 4 vehicles, used only by the public, or categorized as restricted production roads or vehicle track), or high-traffic roads (≥1 vehicle/week).

## Results

### Mammals in the Study Area

We detected 28 species of large and medium-sized mammals through visual observation (398 km walked) or camera trapping (3132 trapping days). We aggregated 23 of them in 17 groups for data analyses (Table 1 & Supporting Information). Because most of the groups were monospecific, we refer to them hereafter as species. We suspected 5 other species were present, but indirect signs of their presence may have been falsely attributed to species confirmed present. Thus, we aggregated these suspected species into groups confirmed present. We included *Cephalophus callipygus* and *Cephalophus leucogaster* in the red duiker group and *Herpestes sanguineus*, *Crossarchus platycephalus*, and *Bdeogale nigripes* in the group of small terrestrial carnivores. The following 10 species were not included in the analyses due to rare or equivocal observations: hippopotamus, leopard, mustelids (*Aonyx congicus*, *Lutra maculicollis*), small arboreal carnivores (*Genetta maculata*, *Genetta servalina*, *Nandinia binotata*), and some monkeys (*Mandrillus sphinx*, *Colobus satanas*, *Lophocebus albigena*).

### Associations of Mammal Abundance or Presence with Disturbances

For each species, we evaluated AIC values from between 153 and 231 candidate models and 3–9 covariates remained in the step 1 final models (Supporting Information). Among final candidate models, there was no spatial autocorrelation in the residuals. For each species, results of our sensitivity analysis showed coefficients and standard errors for the general road variable were comparable among the 20 step-1 models with lowest AIC. This result suggests the associations we found are robust.

The distribution variable was significantly associated (*p* < 0.05) with the general road variable only for buffalo, although the distribution variables of 10 of the 17 species were significantly associated with at least one road characteristic (Table 2). Characteristics related to main roads had significant negative associations with distribution variables of buffalo, gorilla, and sitatunga and positive associations with the abundance of red river hog (*Potamochoerus porcus*). Secondary road characteristics had significant negative associations with distribution variables of elephant and small terrestrial carnivores and positive associations with distribution variables of chimpanzee and red duikers. High levels of traffic had a negative association with distribution variables of elephants, buffalo, and chimpanzee and a positive association with distribution variables of red river hog, small terrestrial carnivores, collared mangabey (*Cercocebus torquatus*), and spot-nosed monkey. For collared mangabey and small terrestrial carnivores, road characteristics associated with main roads had both positive and negative associations with the distribution variable. Secondary road characteristics had similarly equivocal associations for red river hog and collared mangabey. Restricted industrial roads had a positive association with the distribution variable of elephant.

**Table 2:** Significant associations (*p* ≤ 0.05) between road and other human-disturbance variables and abundance or presence of 17 mammal species or groups of species and size of the effect

Species or group of species^a^	Factor^b^	Factor^b^ effect^c^	Variable^d^	Variable unit^e^	Odds ratio or incident rate ratio (95% CI)^f^	*p*
Road variables						
Forest elephant	high traffic	−	DMTRd	km	1.438 (1.130–1.831)	0.003
			DVLTRd	km	0.796 (0.659–0.962)	0.018
	secondary roads	−g	DDgRd	km	1.305 (1.062–1.604)	0.011
			DPubRd	km	1.256 (1.041–1.516)	0.017
			D4×4Rd	km	1.412 (1.017–1.961)	0.040
			DSdRd	km	1.405 (1.004–1.967)	0.047
			DRPdRd	km	0.875 (0.767–0.999)	0.048
Buffalo	roads	−	LRd2	km/km^2^	0.582 (0.340–0.998)	0.049
			L2MdRd	km/km^2^	0.121 (0.037–0.398)	0.001
	main roads	−	L2TrRd	km/km^2^	0.111 (0.018–0.666)	0.016
			L2CoURd	km/km^2^	0.237 (0.059–0.954)	0.043
	high traffic	−	L2VHTRd	km/km^2^	0.267 (0.083–0.856)	0.026
Sitatunga	main roads	−	DCarRd	km	1.410 (1.096–1.814)	0.008
Yellow-backed duiker	nsa^h^					
Blue Duiker	nsa^h^					
Red duikers	secondary roads	+	DSdRd	km	0.446 (0.203–0.983)	0.045
Red river hog	main roads	+	DCarRd	km	0.831 (0.748–0.924)	0.001
			DTrkRd	km	0.901 (0.840–0.966)	0.004
			DLgRd	km	0.910 (0.830–0.997)	0.044
	high traffic	+	DHTRd	km	0.818 (0.716–0.934)	0.003
	secondary roads	+	DPubRd	km	0.899 (0.819–0.986)	0.025
		−	DDgRd	km	1.111 (1.011–1.222)	0.029
Water chevrotain	nsa^h^					
Side-striped jackal	nsa^h^					
Small terrestrial carnivores	main roads	+	L2LtRd	km/km^2^	8.585 (2.930–25.150)	<0.001
			L2CPURd	km/km^2^	5.114 (1.645–15.899)	0.005
			L2PrdRd	km/km^2^	19.708 (2.277–170.538)	0.007
			L2GdRd	km/km^2^	2.664 (1.249–5.680)	0.011
		−	L2TrRd	km/km^2^	0.139 (0.025–0.770)	0.024
	high traffic	+	L2HTRd	km/km^2^	6.679 (1.921–23.219)	0.003
	secondary roads	−	L24×4Rd	km/km^2^	0.081 (0.010–0.668)	0.020
			L2BdRd	km/km^2^	0.125 (0.016–0.944)	0.044
Western lowland gorilla	main roads	−	DPrdRd	km	4.707 (1.547–14.320)	0.006
Central African chimpanzee	high traffic	−	DHTRd	km	1.658 (1.146–2.397)	0.007
			DVLTRd	km	0.616 (0.430–0.881)	0.008
	secondary roads	+	DDgRd	km	0.722 (0.527–0.987)	0.041
Collared mangabey	secondary roads	+	DVTkRd	km	0.487 (0.328–0.723)	<0.001
			DDgRd	km	0.750 (0.578–0.973)	0.030
		−	DRPdRd	km	1.322 (1.065–1.643)	0.012
	high traffic	+	DMTRd	km	0.693 (0.547–0.878)	0.002
	main roads	−	DMjRd	km	1.442 (1.044–1.991)	0.026
		+	DPrdRd	km	0.785 (0.619–0.997)	0.047
Spot-nosed monkey	high traffic	+	L5MTRd	km/km^2^	9.309 (1.831–47.342)	0.007
Common monkeys	nsa^h^					
Brushed-tailed porcupine	nsa^h^					
Giant-pouched rat	nsa^h^					
Other human-disturbance variables						
Forest elephant	settlements	−	DGamba	km	1.088 (1.038–1.142)	<0.001
Buffalo	highly disturbed sector	−	Sct	level	2.863 (1.510–5.431)	0.001
Sitatunga	agriculture	+	TLCT	level	17.832 (1.468–216.597)	0.024
	industries	+	DInd	km	0.942 (0.890–0.997)	0.041
Yellow-backed duiker	nsa^h^					
Blue Duiker	highly disturbed sector	−	Sct	level	3.662 (1.258–10.659)	0.017
	industries	−	DInd	km	1.065 (1.004–1.129)	0.036
Red duikers	agriculture	−	DNPl	km	1.796 (1.189–2.713)	0.005
	industries	+	DInd	km	0.683 (0.520–0.898)	0.006
Red river hog	highly disturbed sector	−	Sct	level	5.646 (2.752–11.583)	<0.001
	agriculture	−	DNPl	km	1.156 (1.067–1.253)	<0.001
	industries	+	DInd	km	0.907 (0.842–0.976)	0.009
	national parks	+	DNPk	km	0.939 (0.892–0.989)	0.017
	settlements	−	DNStl	km	1.070 (1.007–1.137)	0.029
Water chevrotain	nsa^h^					
Side-striped jackal	industries	+	DInd	km	0.784 (0.671–0.915)	0.002
Small terrestrial carnivores	agriculture	+	TLCT	level	37.003 (1.380–992.156)	0.031
Western lowland gorilla	nsa^h^					
Central African chimpanzee	nsa^h^					
Collared mangabey	settlements	−	DNStl	km	1.183 (1.043–1.342)	0.009
	agriculture	−	DNPl	km	1.108 (1.012–1.213)	0.026
Spot-nosed monkey	highly disturbed sector	−	Sct	level	2.672 (1.103–6.477)	0.030
Common monkeys	settlements	−	DNStl	km	1.092 (1.000–1.191)	0.049
Brushed-tailed porcupine	highly disturbed sector	−	Sct	level	3.677 (1.488–9.086)	0.005
	agriculture	+	DNPl	km	0.905 (0.824–0.994)	0.036
	field activities	−	KAIPFA	obs/km	0.584 (0.341–0.999)	0.049
Giant-pouched rat	agriculture	+	DNPl	km	0.549 (0.397–0.759)	<0.001
	industries	−	DInd	km	1.346 (1.102–1.643)	0.004
	highly disturbed sector	−	Sct	level	8.011 (1.507–42.588)	0.015

a Lists of species and scientific names are in Table 1.

b Group of variables as described in the text.

c Sign does not necessarily represent the mathematical sign of the coefficient for each variable: +, variables significantly associated with the factor show a positive association between the disturbances described by the variables and the abundance or presence of the species; –, variables significantly associated with the factor show a negative association between the disturbances described by the variables and the abundance or presence of the species. If inconsistency exists among variables, both signs are noted.

d Abbreviations: D4×4Rd, distance to nearest road used by 4×4 vehicles; DCarRd, distance to nearest road used by cars; DDgRd, distance to nearest degraded road; DGamba, distance to Gamba; DHTRd, distance to nearest high- traffic road; DInd, distance to nearest industrial site; DLgRd, distance to nearest large road; DMjRd, distance to nearest major road; DMTRd, distance to nearest medium traffic road; DNPl, distance to *t* nearest plantation; DNStl, distance to nearest human settlement; DPrdRd, distance to nearest production road; DPubRd, distance to nearest public road; DRPdRd, distance to nearest restricted production road; DSdRd, distance to nearest sand road; DTrkRd, distance to nearest road used by trucks; DVLTRd, distance to nearest very low traffic road; DVTkRd, distance to nearest vehicle tracks; KAIPFA, kilometric abundance index for people's field activities; L24×4Rd, density of roads used by 4×4 vehicles within 2.1 km; L2BdRd, density of bad roads within 2.1 km; L2CoURd, density of medium-width roads within 2.1 km; L2CPRd, density of roads used by corporate and the general public within 2.1 km; L2GdRd, density of good roads within 2.1 km; L2HTRd, density of high-traffic roads within 2.1 km; L2LtRd, density of laterite roads within 2.1 km; L2MdRd, density of medium-width roads within 2.1 km; L2PrdRd, density of production roads within 2.1 km; L2TrRd, density of tar roads within 2.1 km; L2VHTRd, density of very-high-traffic roads within 2.1 km; L5MTRd, density of medium-traffic roads within 0.5 km; LRd2, density of roads within 2.1 km; Sct, sector; TLCT, transect land-cover type (Supporting Information).

e Units: kilometer, distance; kilometers per square kilometer, density; level, category; number of observations per kilometer sampled (obs/km), count.

f Odds ratios: factor by which the expected probability of presence increases when the value of the covariate increases by one unit. Incident rate ratio: factor by which the count distribution variable increases when the value of the covariate increases by one unit.

g We considered elephant presence negatively associated with secondary road factors even though elephant presence was positively associated with production roads under restricted access policies (see text).

h No significant association (p > 0.05) of the road or other human disturbance variables with the distribution variable in this species or group of species in steps 2 and 3 final models.

**Figure 2:**
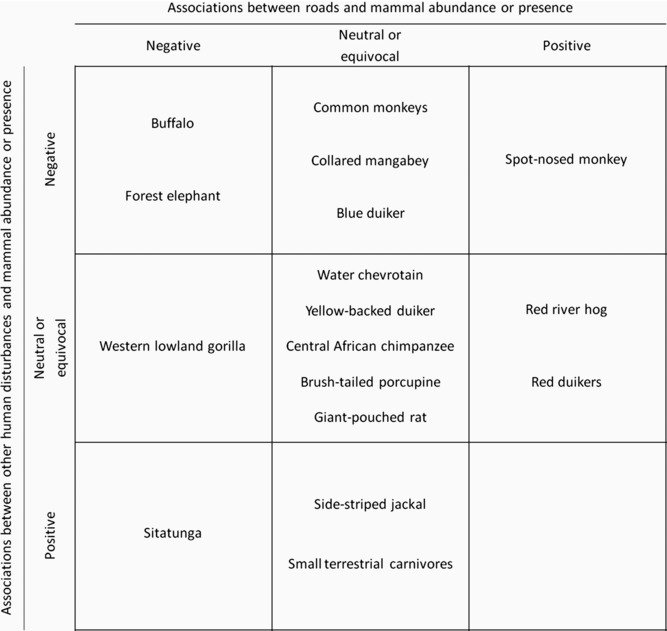
Diversity of associations between roads, other human disturbances, and abundance or presence for 17 species or groups of species of mammals. Negative association indicates species abundance or presence was only negatively associated with roads or other human-disturbance factors (Table 2). Positive association indicates species abundance or presence was only positively associated with roads or other human-disturbance factors, or species abundance or presence was at least positively associated with main roads or high-traffic factors (Table 2). Neutral association indicates species distribution was not significantly associated with roads or other human-disturbance variables (all p > 0.05). Equivocal associations are all other cases.

Overall, the distribution variables of buffalo, elephant, gorilla, and sitatunga were negatively associated with roads (Fig. 2), whereas the distribution variables of collared mangabey and chimpanzee were positively associated with characteristics of secondary roads and negatively associated with characteristics of main roads. Distribution variables of spot-nosed monkey (*Cercopithecus nictitans*) and red duikers were positively associated with roads. The distribution variable of red river hog was positively associated with main-road and high-traffic characteristics and negatively associated with secondary-road characteristics and was thus considered positively associated with roads overall (Fig. 2). No road variable was significantly associated with the abundance or presence of common monkeys, blue duiker (*Philantomba monticola*), yellow-backed duiker (*Cephalophus silvicultor*), water chevrotain (*Hyemoschus aquaticus*), side-striped jackal (*Canis adustus lateralis*), brush-tailed porcupine (*Atherurus africanus*), and giant pouched rat (*Cricetomys emini*).

The distribution variables of sitatunga, jackal, and small terrestrial carnivores were positively associated with other human disturbances. No other human disturbance was significantly associated with distribution variables of gorilla, yellow-backed duiker, water chevrotain, or chimpanzee. The association of the distribution variables of brush-tailed porcupine, giant-pouched rat, red river hog, and red duikers and other human disturbances was equivocal. Some other human disturbances were positively associated and some negatively associated with the distribution variable for the same species. Agriculture was positively associated with the distribution variables of sitatunga, small terrestrial carnivores, and rodents and negatively associated with the distribution variables of red duikers, red river hog, and collared mangabey (Table 2). Proximity to industries was positively associated with the distribution variables of sitatunga, red river hog, jackal, and red duikers and negatively associated with distribution variables of blue duiker and giant-pouched rat. Location in the highly disturbed sector and proximity to settlements were only negatively and significantly associated with the distribution variables of 9 species (Table 2).

## Discussion

We documented the association between different characteristics of roads and abundance or presence of a community of mammals. In our statistical analyses we evaluated approximately 200 models for each species, which may have led to the inclusion of spurious effects. Furthermore, the road variables incorporated in step 2 of the analyses may act as partial surrogates for unmodeled effects of the environment (Crawley 2007). We also acknowledge that, even though we detected no collinearity in the final models, some of our covariates were not independent. For these reasons, as in all observational studies, we cannot infer any causative relations between the covariates and the distribution variables. Furthermore, because we modeled the effects of human activities and ecological factors on the distribution of a broad suite of mammal species, we necessarily omitted attributes of individuals (e.g., age, sex, social status) that affect space use and foraging (Bolnick et al. 2003; Bowyer 2004; Main 2008). Therefore, even when our results suggested that human activities did not affect the distribution of some species, the lack of accounting for these subgroups in modeling could hide strong associations (e.g., Colchero et al. 2010; Conde et al. 2010; van Toor et al. 2011).

The use of camera traps made it possible to document more species than in any comparable study in central Africa (e.g., Blom et al. 2005; Laurance et al. 2008; Van Vliet & Nasi 2008), and our study design enabled us to document significant associations between the roads and abundance or presence of species such as elephant, buffalo, red river hog, and gorilla that were not found in other studies (Blom et al. 2004; Van Vliet & Nasi 2008). Ours is the first African study to document the size and uncertainty of the apparent effects of roads and other human disturbances on abundance or presence of mammal species.

Negative associations between roads and other human disturbances and the abundance or presence of elephant, monkeys, and blue duiker have been reported (e.g., Barnes et al. 1991; Blom et al. 2005; Buij et al. 2007). In our study, abundance or presence of 8 species was negatively associated with at least one characteristic of roads, and for 10 species it was negatively associated with at least one other type of human disturbance (Table 2), which suggests hunting is a major driver of mammal distribution. Proximity to settlements was consistently negatively associated with mammal abundance or presence, probably because hunting tends to decrease as distance to settlements increases (Peres 2000) and roads facilitate access of hunters to otherwise inaccessible areas (Wilkie et al. 2000; Geist & Lambin 2002).

In our site, roads with certain characteristics were negatively associated with distribution variables of elephant, buffalo, gorilla, chimpanzee, and sitatunga, which are considered flagship species for conservation and tourism in Gabon. Except for elephant and buffalo, these species were not negatively associated with other human disturbances. This result suggests these species are more sensitive to roads than to other human disturbances. This likely results from their relatively low reproductive rates (Rytwinski & Fahrig 2011) and because their size forces poachers to hunt the animals close to a road so they can transport the meat in vehicles.

Our results also differed from those of other researchers. For example, abundances of duikers and diurnal monkeys are negatively associated with road presence in other sites (e.g., Blom et al. 2005; Laurance et al. 2008; Van Vliet & Nasi 2008), whereas we found a positive or neutral association. This discrepancy may have resulted because levels of hunting near the roads differed among studies or because the species may avoid roads to a greater extent in mostly forested sites compared with the grassland and forest mosaic at our site. Contrary to the finding of Laurance et al. (2006) in other sites within the Gamba Complex of Protected Areas, we did not detect an association between gorilla presence and human disturbances other than roads, probably because these authors were comparing 2 areas with different levels of hunting, whereas we looked at relative reduction of probability of presence within a hunted area.

Our results suggest a more diverse pattern of responses of large and medium-sized mammals to roads and other human disturbances than has been reported previously (Fig. 2). Some species that probably feed on regrowing vegetation along roadsides, such as red river hogs and red duikers, appear to benefit from the presence of roads. The apparent tolerance of small terrestrial carnivores and jackals to human and road disturbances is probably due to the high adaptability of these species to human disturbances and the apparent lack of interest of hunters in them (H.V., personal observation). Other species, such as the collared mangabey and chimpanzee, apparently avoid main roads but use secondary roads.

A positive association between abundance or presence of mammals and human disturbance indicates species find resources associated with the disturbance. For example, sitatunga and rodents are probably attracted by crops, and small terrestrial carnivores are probably preying on these rodents. Surprisingly, in our results elephant distribution did not seem affected by distance to plantations, although crop raiding is a major issue for local human populations at our site and others in the region (Barnes 1996). Plantations are thus probably not a substantial food source for elephants in our area.

Another emerging pattern from our results that could inform wildlife management is the possible role of industrial concessions as refuges. Industrial sites were positively associated with the abundance or presence of sitatunga, red duikers, red river hog, and jackal, probably because they provide protection from hunters. Furthermore, results of our study and of Kolowski et al.'s (2010) study suggest elephant abundance around industrial roads with restricted access can be high, probably because hunting is limited and roads serve as movement corridors. These findings are consistent with the idea that industrial concessions are effective in protecting flagship species when hunting is restricted and driving regulations (40 km/h speed limit and no driving at night) are enforced, as occurs on restricted production roads in our site (Laurance et al. 2006; Kolowski et al. 2010). Nevertheless, our results showed an opposite association for blue duiker and brush-tailed porcupine, which suggests that the protection afforded by industrial sites may be limited to larger animals and that poaching continues in these areas for smaller species.

In terms of management options, direct effects of roads on mammals (e.g., road avoidance, fragmentation and edge effects, use of roads as movement corridors, and resource gain from regrowing vegetation along roads) can be monitored by centering animal sampling on the road network, and mitigation options are adjustments to the road design. By contrast, indirect effects of roads on mammals (e.g., hunting, agriculture, and urbanization along roads) must be monitored by systematic sampling of animals in the area and surveys of social and economic drivers of the disturbances, and mitigation options involve participatory planning of human development. In a relatively undisturbed area with a new road network it would likely be more effective to focus on direct effects. By contrast, in our disturbed study area with a 50-year-old network of roads and coincident human development, focusing mitigation measures on indirect effects of roads and on other human disturbances likely would be more effective because construction of roads is probably a companion process to human development (Wilkie et al. 2000; Geist & Lambin 2002). Therefore, we suggest management of the area include actions that concentrate agriculture in zones that are already disturbed and where environmentally sustainable practices can be promoted (e.g., extend duration of crop rotations, ensure trees that serve as sources of recruitment are not eliminated), control illegal hunting, limit access to existing roads (i.e., no unauthorized farmers and settlers or illegal hunters), integrate future road construction in a general development plan for the region, and consider the possible role of industrial concessions as mammal refuges.

## Supporting Information

A list of all variables used in the modeling process, with acronyms, units, and methods of estimation and calculation (Appendix S1), models with lowest AIC for each species in step 1 of the modeling process (Appendix S2), and parameters, AIC, effect sizes, and *p* values of final models for each species (Appendix S3) are available online. The authors are solely responsible for the content of these materials. Queries (other than absence of the material) should be directed to the corresponding author.

## Supplementary material

Disclaimer: Supplementary materials have been peer-reviewed but not copyedited.
