# Supplementary material for: Distribution of a Community of Mammals in Relation to Roads and Other Human Disturbances in Gabon, Central Africa
Source: Conserv Biol. 2013 Feb 14;27(2):281–91. doi: 10.1111/cobi.12017 (PMC3644169; doi:10.1111/cobi.12017)
Supplement: Supplementary file 1 [file cobi0027-0281-SD1.pdf]

Table S1: list of all variables used in the modeling process, with acronyms, units, and methods of estimation and calculation.

Acronym	*description (units)*	*method of estimation and calculation*
Abundance variables		
CST	Count of signs on transect: quantitative discrete (observations).	We counted the number of observations of signs of mammals on transects. Signs included, aggregation of species, season and mode of calculation are described in table 1 and the rationale in footnote [a].
CTE	Count of trapping events: quantitative discrete (trapping events).	We counted the number of individuals (based on time between events and individual characteristics) photographed by the camera trap on the transects. Aggregation of species, season and mode of calculation are described in table 1 and the rationale in footnote [a].
PA	Presence-absence: binary (0 or 1)	We assigned sampling units to '1' if CST≥1 or CTE≥1; to '0' otherwise. The use of CST, CTE or both, the signs included, aggregation of species, season and mode of calculation are described in table 1 and the rationale in footnote [a].
Ecosystem variables		
Season	Season: qualitative nominal (wet or dry)	We assigned sampling units to 'wet' if monitored between November 2010 and January 2011; to 'dry' if monitored between June and August 2011. Season was not included in the analysis for some species when wet and dry season data was pooled.
TVi	Transect visibility: qualitative ordinal (very high-5 > high-4 > medium-3 > low-2 > very low-1).	We scored visibility of animals on the transect visually for each 100 m section of the transect between 1 (very low visibility) and 5 (high visibility). TVi is the rounded average of visibility scores for the 5 sections.
DEco	Distance to the nearest ecotone: quantitative continuous (km).	We calculated with a geographical information system (GIS) the distance (straight line) from the transect midpoint to the nearest Forest/Savanna, or Forest/Swamp, or Savanna/Swamp interface in meters.
LEct2	Density of ecotones within 2.1 km from the transect: quantitative continuous (km/km²).	We calculated with a GIS the average length of ecotones per square kilometer within a 2.1 km buffer (15.95 km²) around the transect line.
LEct5	Density of ecotones within 0.5 km from the transect: quantitative continuous (km/km²).	We calculated with a GIS the average length of ecotones per square kilometer within a 0.5 km buffer (1.29 km²) around the transect line.
TLCT	Transect land cover type: qualitative nominal (permanent swamp, periodically inundated, old secondary, young secondary, gallery, coastal vegetation, old plantation,	We estimated dominant land cover type visually for each 100 m section of the transect. TLCT is the most represented modality of dominant land cover types in the 5 sections.

	grassland).	
TUT	Transect understory type: qualitative nominal (shrub, liana, marantaceae, fern, grass, bare soil).	We estimated dominant understory type visually for each 100 m section of the transect. TUT is the most represented modality of dominant understory types in the 5 sections.
TCS	Transect canopy score: quantitative discrete (6-10=continuous > 1-5=discontinuous > 0=no canopy).	We scored canopy cover visually for each 100 m section of the transect between 0 (no canopy) and 2 (closed canopy). TCS is the sum of the canopy cover scores for the 5 sections.
TIS	Transect inundation score: quantitative discrete (6-10=inundated > 2-5=partially inundated > 0-1=dry).	We scored ground wetness visually for each 100 m section of the transect between 0 (dry) and 2 (inundated). TIS is the sum of the ground wetness scores for the 5 sections.
TTP	Transect topographic profile: quantitative discrete (>7=very hilly > 2-7=hilly > 0-1=flat).	We scored topographic profile visually for each 100 m section of the transect (ridge or plateau-0, gully bottom-1, slope-2, high slope-3). TTP is the sum of the topographic scores for the 5 sections.
DFWater	Distance to the nearest fresh water source: quantitative continuous (km).	We calculated with a GIS the distance (straight line) from the transect midpoint to the nearest lagoon, lake, river or stream.
DSwamp	Distance to the nearest swamp zone: quantitative continuous (km).	We calculated with a GIS the distance (straight line) from the transect midpoint to the nearest swamp zone.
DOcean	Distance to the ocean: quantitative continuous (km).	We calculated with a GIS the distance (straight line) from the transect midpoint to the nearest ocean shore.
Human disturbance variables other than road		
Sct	Sector: qualitative nominal (highly disturbed sector or lightly disturbed sector).	We assigned sampling units to 'highly disturbed sector' if situated in the corresponding area; to 'lightly disturbed sector' otherwise.
DNPl	Distance to the nearest plantation: quantitative continuous (km).	We calculated with a GIS the distance (straight line) from the transect midpoint to the nearest plantation and plantation camp.
KAI_{PFA}	Kilometric abundance index for people's field activities: quantitative discrete (obs./km).	We recorded all signs of presence of humans on transects (including public roads, vehicle tracks, pedestrian paths and trails, used bush rifle cartridges, gunshots heard, cable snares, direct observations, and felled trees). KAI_{PFA} is the number of such observations per kilometer of transect walked. Depending on the group considered, KAI_{PFA} was calculated for each season or for the entire study.
DNStl	Distance to the nearest human settlement: quantitative continuous (km).	We calculated with a GIS the distance (straight line) from the transect midpoint to the nearest village, town, or oil company camp listed in the database.
DGamba	Distance to Gamba town: quantitative continuous (km).	We calculated with a GIS the distance (straight line) from the transect midpoint to the center of the Gamba town.
DInd	Distance to the nearest	We calculated with a GIS the distance (straight line) from

	industrial site: quantitative continuous (km).	the transect midpoint to the nearest industrial building, surface pipeline, or active wells (oil or water) listed in the database. We did not consider inactive wells or buried pipelines.
KAI_{IA}	Kilometric abundance index for industrial activity: quantitative discrete (obs./km).	We recorded all signs of presence of industrial activities on transects (including major and production roads, pipelines, laterite and gravel extraction, and abandoned industrial equipment). KAI_{IA} is the number of such observations per kilometer of transect walked. Depending on the group considered, KAI_{IA} was calculated for each season or for the entire study.
DNPk	Distance to the nearest national park: quantitative continuous (km).	We calculated with a GIS the distance (straight line) from the transect midpoint to the nearest national park including Moukalaba-Doudou and Loango national parks.
General road variables – general assessment		
DRd	Distance (straight line) to the nearest road: quantitative continuous (km).	We recorded the location of all roads in the study area using a GPS device. We calculated with a GIS the distance (straight line) from the transect midpoint to the nearest road listed in the database, whatever type.
LRd2	Density of roads within 2.1 km from the transect: quantitative continuous (km/km²).	We recorded the location of all roads in the study area using a GPS device. We calculated with a GIS the average length of roads (whatever type) per square kilometer in a 2.1 km buffer (15.95 km²) around the transect line.
LRd5	Density of roads within 0.5 km from the transect: quantitative continuous (km/km²).	We recorded the location of all roads in the study area using a GPS device. We calculated with a GIS the average length of ecotones per square kilometer in a 0.5 km buffer (1.29 km²) around the transect line.
Road characteristic variables – road characteristic assessment<br>*DRd family*		
DTrRd<br>DLtRd<br>DSdRd	Distances to the nearest road for each coating category: quantitative continuous (km).	We recorded visually the type of coating (tar, laterite, or sand) of all roads in the study area. We calculated with a GIS the distance (straight line) from the transect midpoint to the nearest road for each coating category: 3 resulting variables.
DLrgRd<br>DMdRd<br>DNrwRd	Distances to the nearest road for each rights-of-way category: quantitative continuous (km).	We measured the width of all roads in the study area. We calculated with a GIS the distance (straight line) from the transect midpoint to the nearest road for each rights-of-way category including large (16 to 20m-wide), medium (10 to 15 m-wide) and narrow (≤10m-wide): 3 resulting variables.
DGdRd<br>DDgRd<br>DBdRd	Distances to the nearest road for each condition category: quantitative continuous (km).	We scored visually the condition of all roads (good, degraded, or bad) in the study area. We calculated with a GIS the distance (straight line) from the transect midpoint to the nearest road for each condition category: 3 resulting variables.
DTrkRd<br>DCarRd	Distances to the nearest road for each vehicle category:	We recorded visually the type of vehicle (trucks, cars, or ATV) able to use the roads in the study area. We

D4x4Rd	quantitative continuous (km).	calculated with a GIS the distance (straight line) from the transect midpoint to the nearest road for each vehicle category: 3 resulting variables.
DVHTRd<br>DHTRd<br>DMTRd<br>DLTRd<br>DVLTRd	Distances to the nearest road for each traffic category: quantitative continuous (km).	We estimated the annual intensity of traffic on all roads in the study area with direct counts of encounters and interviews of road users and regulators. We calculated with a GIS the distance (straight line) from the transect midpoint to the nearest road for each traffic category including very high (≥11 veh./day:), high (2 to 10 veh./day:), medium (1 to 7 veh./week:), low (1 to 4 veh./month:), and very low (<1 veh./month:): 5 resulting variables.
DCoURd<br>DPuURd<br>DCPURd	Distances to the nearest road for each category of use: quantitative continuous (km).	We estimated the dominant type of people driving on each road in the study area with interviews of road users and regulators. We calculated with a GIS the distance (straight line) from the transect midpoint to the nearest road for each category of use including corporate (employees of the oil company and contractors for work purposes), general public (inhabitants of the area for non-working purposes), and both: 3 resulting variables.
DMjRd<br>DPrdRd<br>DRPdRd<br>DPubRd<br>DVTkRd	Distances to the nearest road for each road type: quantitative continuous (km).	Using the above characteristics of roads[b], we categorized all roads in the area into 5 categories including major, production, restricted production, public, and vehicle track. We calculated with a GIS the distance (straight line) from the transect midpoint to the nearest road for each road type: 5 resulting variables.
LRd2 family		
L2TrRd<br>L2LtRd<br>L2SdRd	Density of roads within 2.1 km from the transect for each coating category: quantitative continuous (km/km²).	We recorded visually the type of coating (tar, laterite, or sand) of all roads in the study area. We calculated with a GIS the average length of roads per square kilometer in a 2.1 km-radius surface (15.95 km²) around the transect line for each coating category: 3 resulting variables.
L2LrgRd<br>L2MdRd<br>L2NrwRd	Density of roads within 2.1 km from the transect for each rights-of-way category: quantitative continuous (km/km²).	We measured the width of all roads in the study area. We calculated with a GIS the average length of roads per square kilometer in a 2.1 km-radius surface (15.95 km²) around the transect line for each rights-of-way category including large (16 to 20m-wide), medium (10 to 15 m-wide) and narrow (≤10m-wide): 3 resulting variables.
L2GdRd<br>L2DgRd<br>L2BdRd	Density of roads within 2.1 km from the transect for each condition category: quantitative continuous (km/km²).	We scored visually the condition of all roads (good, degraded, or bad) in the study area. We calculated with a GIS the average length of roads per square kilometer in a 2.1 km-radius surface (15.95 km²) around the transect line for each condition category: 3 resulting variables.
L2TrkRd<br>L2CarRd<br>L24x4Rd	Density of roads within 2.1 km from the transect for each vehicle category: quantitative continuous (km/km²).	We recorded visually the type of vehicle (trucks, cars, or ATV) able to use the roads in the study area. We calculated with a GIS the average length of roads per square kilometer in a 2.1 km-radius surface (15.95 km²)

		around the transect line for each vehicle category: 3 resulting variables.
L2VHTRd<br>L2HTRd<br>L2MTRd<br>L2LTRd<br>L2VLTRd	Density of roads within 2.1 km from the transect for each traffic category: quantitative continuous (km/km²).	We estimated the annual intensity of traffic on all roads in the study area with direct counts of encounters and interviews of road users and regulators. We calculated with a GIS the average length of roads per square kilometer in a 2.1 km-radius surface (15.95 km²) around the transect line for each traffic category including very high (≥11 veh./day:), high (2 to 10 veh./day:), medium (1 to 7 veh./week:), low (1 to 4 veh./month:), and very low (≤1 veh./month:): 5 resulting variables.
L2CoURd<br>L2PuURd<br>L2CPURd	Density of roads within 2.1 km from the transect for each category of use: quantitative continuous (km/km²).	We estimated the dominant type of people driving on each road in the study area with interviews of road users and regulators. We calculated with a GIS the average length of roads per square kilometer in a 2.1 km-radius surface (15.95 km²) around the transect line for each category of use including corporate (employees of the oil company and contractors for work purposes), general public (inhabitants of the area for non-working purposes), and both: 3 resulting variables.
L2MjRd<br>L2PrdRd<br>L2RPdRd<br>L2PubRd<br>L2VTkRd	Density of roads within 2.1 km from the transect for each road type: quantitative continuous (km/km²).	Using the above characteristics of roads[b], we categorized all roads in the area into 5 categories including major, production, restricted production, public, and vehicle track. We calculated with a GIS the average length of roads of each type per square kilometer in a 0.5 km-radius surface (1.29 km²) around the transect line for each road type: 5 resulting variables.
LRd5 family		
L5TrRd<br>L5LtRd<br>L5SdRd	Density of roads within 0.5 km from the transect for each coating category: quantitative continuous (km/km²).	We recorded visually the type of coating (tar, laterite, or sand) of all roads in the study area. We calculated with a GIS the average length of roads per square kilometer in a 0.5 km buffer (1.29 km²) around the transect line for each coating category: 3 resulting variables.
L5LrgRd<br>L5MdRd<br>L5NrwRd	Density of roads within 0.5 km from the transect for each rights-of-way category: quantitative continuous (km/km²).	We measured the width of all roads in the study area. We calculated with a GIS the average length of roads per square kilometer in a 0.5 km buffer (1.29 km²) around the transect line for each rights-of-way category including large (16 to 20m-wide), medium (10 to 15 m-wide) and narrow (≤10m-wide): 3 resulting variables.
L5GdRd<br>L5DgRd<br>L5BdRd	Density of roads within 0.5 km from the transect for each condition category: quantitative continuous (km/km²).	We scored visually the condition of all roads (good, degraded, or bad) in the study area. We calculated with a GIS the average length of roads per square kilometer in a 0.5 km buffer (1.29 km²) around the transect line for each condition category: 3 resulting variables.
L5TrkRd<br>L5CarRd<br>L54x4Rd	Density of roads within 0.5 km from the transect for each vehicle category: quantitative	We recorded visually the type of vehicle (trucks, cars, or ATV) able to use the roads in the study area. We calculated with a GIS the average length of roads per

	continuous (km/km²).	square kilometer in a 0.5 km buffer (1.29 km²) around the transect line for each vehicle category: 3 resulting variables.
L5VHTRd<br>L5HTRd<br>L5MTRd<br>L5LTRd<br>L5VLTRd	Density of roads within 0.5 km from the transect for each traffic category: quantitative continuous (km/km²).	We estimated the annual intensity of traffic on all roads in the study area with direct counts of encounters and interviews of road users and regulators. We calculated with a GIS the average length of roads per square kilometer in a 0.5 km buffer (1.29 km²) around the transect line for each traffic category including very high (≥11 veh./day:), high (2 to 10 veh./day:), medium (1 to 7 veh./week:), low (1 to 4 veh./month:), and very low (≤1 veh./month:): 5 resulting variables.
L5CoURd<br>L5PuURd<br>L5CPURd	Density of roads within 0.5 km from the transect for each category of use: quantitative continuous (km/km²).	We estimated the dominant type of people driving on each road in the study area with interviews of road users and regulators. We calculated with a GIS the average length of roads per square kilometer in a 0.5 km buffer (1.29 km²) around the transect line for each category of use including corporate (employees of the oil company and contractors for work purposes), general public (inhabitants of the area for non-working purposes), and both: 3 resulting variables.
L5MjRd<br>L5PrdRd<br>L5RPdRd<br>L5PubRd<br>L5VTkRd	Density of roads within 0.5 km from the transect for each road type: quantitative continuous (km/km²).	Using the above characteristics of roads[b], we categorized all roads in the area into 5 categories including major, production, restricted production, public, and vehicle track. We calculated with a GIS the average length of roads of each type per square kilometer in a 0.5 km-radius surface (1.29 km²) around the transect line for each road type: 5 resulting variables.

[a] We calculated one abundance variable for each species or group of species and for each transect using the following rationale:

1. We grouped together taxonomically-related species occupying a comparable ecological niche when:
    - the number of observations (counts of signs on the transects and trapping events) was insufficient for the species (less than 10 or less than 10% of transects having at least one observation);
    - the majority of signs could not be assigned to a unique species (red duikers and small terrestrial carnivores); or
    - the ability of detecting a species was related to the detection of another (monkeys).
2. We grouped wet and dry season observations in a single variable or discarded data from one sector or land cover type when:
    - the number of observations (including counts of signs on the transects and trapping events) were less than 10 for all transects or less than 10% of transects had at least one observation within a season, sector or land cover type; or
    - a bias in the detectability of signs was suspected between season, sector or land cover type.

3. We included as many types of signs as possible into the resulting abundance variables.
    - We used the counts of trapping events alone when less than 10% of transects had at least one observation on transects (blue duiker and giant-pouched rat).
    - We used both counts of observations on transects and counts of trapping events in presence-absence variables when only the forest land cover type was considered (yellow-backed duiker, red duikers, water chevrotain, gorilla, chimpanzee, collared mangabey, and brush-tailed porcupine).
    - We used only counts of observations on transects when:
        - o both forest and savanna land cover type were considered (elephant, buffalo, sitatunga, and red river hog);
        - o only the savanna land cover type was considered (side-striped jackal and small terrestrial carnivores); or
        - o the number of camera trapping events was less than 10 on all transects or less than 10% of transects had at least one trapping event (spot-nosed monkey and all monkeys).
4. We used the number of individuals instead of the number of groups of mammals to calculate the variable when:
    - a species was solitary or foraging in small groups (duikers, water chevrotain);
    - signs can lead to a reliable estimation of the number of individuals in the group (apes); or
    - only direct observations or camera trapping events are available for estimating the variable (blue duiker, brush-tailed porcupine, and giant-pouched rat).
5. Finally, some resulting count variables were transformed into presence-absence variables when:
        - o more than 90% of transects had either 0 or 1 sign (yellow-backed duiker); or
        - o estimating the number of groups in the field was impossible due to the over-abundance of signs (elephant).

[b] Combination of road characteristics and main road types considered in the study.

Type of road	*coating*	*rights of way*	*condition*	*vehicles*	*traffic*	*use*	*restricted access*
Major	tar or laterite	large	variable	trucks	very high or high	both	no
Production	laterite	large or medium	good	trucks	variable	corporate	no
Restricted production	laterite	large or medium	good	trucks	variable	corporate	yes
Public	variable	variable	variable	variable	variable	public	no
Vehicle tracks	sand	variable	bad	All terrain vehicles	low or very low	public	no
