# Supplementary material for: Distribution of a Community of Mammals in Relation to Roads and Other Human Disturbances in Gabon, Central Africa
Source: Conserv Biol. 2013 Feb 14;27(2):281–91. doi: 10.1111/cobi.12017 (PMC3644169; doi:10.1111/cobi.12017)
Supplement: Supplementary file 2 [file cobi0027-0281-SD2.pdf]

Table S2: models with lowest AIC during step 1 of modeling process for each species (step 1 final model in grey).

Elephant

Model	*AIC*	*ΔAIC*	*AIC weights*
PA ~ TVi + Season + LEct5 + DOcean + DSwamp + DGamba + KAIIA	367,8	0,0	79,8%
PA ~ TVi + Season + LEct5 + TCS + DOcean + DSwamp + KAIPFA + DGamba + KAIIA	368,3	0,5	6,6%
PA ~ TVi + Season + DEco + LEct5 + TCS + DOcean + DSwamp + KAIPFA + DGamba + KAIIA	368,4	0,6	4,0%
PA ~ TVi + Season + LEct5 + TCS + DOcean + DSwamp + DGamba + KAIIA	368,4	0,6	4,0%
PA ~ TVi + Season + LEct5 + TCS + TTP + DOcean + DSwamp + KAIPFA + DGamba + KAIIA	368,6	0,8	1,5%
PA ~ TVi + Season + DEco + LEct5 + DOcean + DSwamp + KAIPFA + DGamba + KAIIA	368,6	0,8	1,5%
PA ~ TVi + Season + LEct5 + DOcean + DSwamp + KAIPFA + DGamba + KAIIA	368,6	0,8	1,5%
PA ~ TVi + Season + DEco + LEct5 + TTP + DOcean + DSwamp + KAIPFA + DGamba + KAIIA	368,7	0,9	0,9%
PA ~ TVi + Season + DEco + LEct5 + TCS + TTP + DOcean + DSwamp + KAIPFA + DGamba + KAIIA	369,0	1,2	0,2%
PA ~ TVi + Season + DEco + LEct5 + TCS + DOcean + DSwamp + DGamba + KAIIA	369,1	1,3	0,1%
PA ~ TVi + Season + LEct5 + DOcean + DSwamp + DGamba	369,3	1,5	0,0%
PA ~ TVi + Season + DEco + LEct5 + TCS + DOcean + DSwamp + KAIPFA + DGamba	369,4	1,6	0,0%
PA ~ TVi + Season + LEct5 + TCS + DOcean + DSwamp + KAIPFA + DGamba	369,4	1,6	0,0%
PA ~ TVi + Season + DEco + LEct5 + TCS + TTP + DOcean + DSwamp + DGamba + KAIIA	369,6	1,8	0,0%
PA ~ TVi + Season + LEct5 + TCS + DOcean + DSwamp + DGamba	369,7	1,9	0,0%
PA ~ TVi + Season + DEco + LEct5 + TCS + TIS + DOcean + DSwamp + KAIPFA + DGamba + KAIIA	369,9	2,1	0,0%
PA ~ TVi + Season + DEco + TCS + DOcean + DSwamp + KAIPFA + DGamba + KAIIA	370,2	2,4	0,0%
PA ~ TVi + Season + DEco + LEct5 + TCS + TTP + DOcean + DSwamp + KAIPFA + DGamba	370,3	2,5	0,0%
PA ~ TVi + Season + LEct5 + TCS + TIS + TTP + DOcean + DSwamp + KAIPFA + DGamba + KAIIA	370,3	2,5	0,0%
PA ~ TVi + Season + DEco + LEct5 + TIS + TTP + DOcean + DSwamp + KAIPFA + DGamba + KAIIA	370,5	2,7	0,0%

Buffalo

Model	*AIC*	*ΔAIC*	*AIC weights*
CST ~ TVi + Season + DEco + TLCT + TIS + DFWater + Sct + DNStl + DInd	509,2	0,0	24,1%
CST ~ TVi + Season + DEco + TLCT + TTP + DFWater + Sct + DNStl + DInd	509,2	0,0	24,1%
CST ~ Season + DEco + TLCT + TIS + DFWater + Sct + DNStl + DInd	509,2	0,0	24,1%
CST ~ Season + DEco + TLCT + DFWater + Sct + DNStl + DInd	509,4	0,2	8,9%
CST ~ TVi + Season + DEco + TLCT + TIS + TTP + DFWater + Sct + DNStl + DInd	509,4	0,2	8,9%
CST ~ TVi + Season + DEco + TLCT + DFWater + Sct + DNStl + DInd	509,5	0,3	5,4%
CST ~ TVi + Season + DEco + LEct2 + TLCT + TIS + DFWater + Sct + DNStl + DInd	509,7	0,5	2,0%
CST ~ Season + DEco + TLCT + TIS + TTP + DFWater + Sct + DNStl + DInd	509,7	0,5	2,0%
CST ~ TVi + Season + DEco + LEct2 + TLCT + TTP + DFWater + Sct + DNStl + Dind	510,1	0,9	0,3%
CST ~ TVi + Season + DEco + LEct2 + TLCT + TIS + TTP + DFWater + Sct + DNStl + DInd	510,4	1,2	0,1%
CST ~ TVi + Season + DEco + TLCT + TIS + TTP + Sct + DNStl + DInd	510,4	1,2	0,1%
CST ~ Season + DEco + LEct2 + TLCT + TIS + TTP + DFWater + Sct + DNStl + DInd	510,6	1,4	0,0%
CST ~ TVi + Season + DEco + TLCT + TIS + Sct + DNStl + DInd	510,8	1,6	0,0%
CST ~ Season + DEco + TLCT + TIS + Sct + DNStl + DInd	510,8	1,6	0,0%
CST ~ TVi + Season + DEco + LEct2 + TLCT + TIS + TTP + Sct + DNStl + DInd	510,9	1,7	0,0%
CST ~ TVi + Season + DEco + TLCT + TIS + TTP + DFWater + Sct + DNPl + DNStl + DInd	511,0	1,8	0,0%
CST ~ Season + DEco + TLCT + DNStl + DInd	511,5	2,3	0,0%
CST ~ TVi + Season + DEco + LEct2 + TLCT + TIS + DFWater + Sct + DNPl + DNStl + DInd	511,5	2,3	0,0%
CST ~ TVi + Season + DEco + LEct2 + TLCT + TIS + TTP + DFWater + Sct + DNPl + DInd	511,6	2,4	0,0%
CST ~ Season + DEco + LEct2 + TLCT + TIS + TTP + DFWater + Sct + DNPl + DNStl + DInd	511,8	2,6	0,0%

Sitatunga

Model	*AIC*	*ΔAIC*	*AIC weights*
CST ~ Season + TLCT + TIS + DFWater + DOcean + DInd	293,5	0,0	24,1%
CST ~ Season + TLCT + DFWater + DOcean + DInd	294,4	0,9	0,3%
CST ~ Season + LEct5 + TLCT + TIS + DFWater + DOcean + Dind	294,4	0,9	0,3%
CST ~ Season + LEct2 + LEct5 + TLCT + TIS + DFWater + DOcean + DInd	294,9	1,4	0,0%
CST ~ Season + LEct5 + TLCT + DFWater + DOcean + Dind	295,0	1,5	0,0%
CST ~ Season + LEct2 + LEct5 + TLCT + TIS + DFWater + DOcean + DSwamp + DInd	295,3	1,8	0,0%
CST ~ Season + TLCT + TIS + DFWater + Docean	295,3	1,8	0,0%
CST ~ Season + LEct5 + TLCT + TIS + DFWater + DOcean + DSwamp + DInd	295,5	2,0	0,0%
CST ~ Season + LEct2 + LEct5 + TLCT + TIS + DFWater + DSwamp + DInd	295,5	2,0	0,0%
CST ~ Season + LEct2 + TLCT + TIS + DFWater + DOcean + DInd	295,5	2,0	0,0%
CST ~ Season + LEct2 + LEct5 + TLCT + DFWater + DOcean + DInd	295,5	2,0	0,0%
CST ~ Season + LEct2 + LEct5 + TLCT + TIS + DFWater + DOcean + KAIPFA + DInd	295,8	2,3	0,0%
CST ~ Season + LEct2 + LEct5 + TLCT + DFWater + DOcean + DSwamp + DInd	295,8	2,3	0,0%
CST ~ Season + LEct2 + LEct5 + TLCT + TIS + DFWater + DOcean + DSwamp + KAIPFA + DInd	296,1	2,6	0,0%
CST ~ Season + LEct2 + LEct5 + TLCT + TIS + DFWater + DSwamp + KAIPFA + DInd	296,1	2,6	0,0%
CST ~ Season + LEct5 + TLCT + TIS + DFWater + DOcean + DSwamp + KAIPFA + DInd	296,4	2,9	0,0%
CST ~ Season + LEct2 + LEct5 + TLCT + DFWater + DOcean + DSwamp + KAIPFA + DInd	296,5	3,0	0,0%
CST ~ Season + LEct5 + TLCT + TIS + DFWater + DOcean	296,5	3,0	0,0%
CST ~ Season + TLCT + DFWater + DOcean	296,5	3,0	0,0%
CST ~ Season + LEct2 + TLCT + TIS + DFWater + DOcean + DSwamp + KAIPFA + DInd	296,7	3,2	0,0%

Yellow-backed duiker

Model	*AIC*	*ΔAIC*	*AIC weights*
PA ~ DOcean	97,1	0,0	24,1%
PA ~ 1	97,1	0,0	24,1%
PA ~ LEct2 + DOcean	98,2	1,1	0,1%
PA ~ DOcean + Sct	98,3	1,2	0,1%
PA ~ LEct2 + DOcean + Sct	98,4	1,3	0,0%
PA ~ LEct2 + Sct	98,4	1,3	0,0%
PA ~ DEco + DOcean + Sct	99,0	1,9	0,0%
PA ~ LEct2	99,0	1,9	0,0%
PA ~ DEco + LEct2 + DOcean + Sct	99,5	2,4	0,0%
PA ~ DEco + LEct2 + DOcean	99,5	2,4	0,0%
PA ~ DEco + DOcean + DSwamp + Sct	99,8	2,7	0,0%
PA ~ LEct2 + DOcean + DSwamp + Sct	99,9	2,8	0,0%
PA ~ DEco + LEct2 + Sct	100,0	2,9	0,0%
PA ~ DEco + LEct2 + DOcean + DSwamp + Sct	100,5	3,4	0,0%
PA ~ DEco + LEct2 + DOcean + DSwamp	100,6	3,5	0,0%
PA ~ DEco + LEct2 + TUT + DOcean + Sct	101,2	4,1	0,0%
PA ~ LEct2 + TUT + DOcean + DSwamp + Sct	101,5	4,4	0,0%
PA ~ DEco + TUT + DOcean + DSwamp + Sct	101,7	4,6	0,0%
PA ~ DEco + LEct2 + TUT + DOcean + DSwamp + Sct	101,9	4,8	0,0%
PA ~ DEco + LEct2 + DSwamp + Sct	102,0	4,9	0,0%

Blue duiker

Model	*AIC*	*ΔAIC*	*AIC weights*
CTE ~ Season + TUT + TIS + DSwamp + Sct + DInd	357,8	0,0	24,1%
CTE ~ TUT + DSwamp	358,6	0,8	0,4%
CTE ~ TUT + DSwamp + Sct + DInd	358,8	1,0	0,2%
CTE ~ Season + TUT + TIS + DSwamp + Sct + DGamba + DInd	359,3	1,5	0,0%
CTE ~ Season + TUT + TIS + Sct + DGamba + DInd	359,3	1,5	0,0%
CTE ~ Season + TUT + DSwamp + Sct + DInd	359,3	1,5	0,0%
CTE ~ Season + TUT + TIS + DSwamp + Sct + DGamba	359,5	1,7	0,0%
CTE ~ Season + DEco + TUT + TIS + DSwamp + Sct + Dind	359,7	1,9	0,0%
CTE ~ TUT + TIS + DSwamp + Sct + DInd	359,8	2,0	0,0%
CTE ~ Season + TUT + TIS + DSwamp + Sct	359,9	2,1	0,0%
CTE ~ TUT + DSwamp + Sct	360,1	2,3	0,0%
CTE ~ Season + TIS + DSwamp + Sct + DInd	360,2	2,4	0,0%
CTE ~ Season + TUT + DSwamp + Sct + DGamba + DInd	360,4	2,6	0,0%
CTE ~ Season + TUT + DSwamp + Sct	360,6	2,8	0,0%
CTE ~ Season + DEco + TUT + TIS + DSwamp + Sct + DGamba + DInd	360,8	3,0	0,0%
CTE ~ Season + TUT + TIS + DSwamp + Sct + DNPl + DGamba + DInd	360,9	3,1	0,0%
CTE ~ TUT + TIS + DSwamp + Sct + DGamba + DInd	361,1	3,3	0,0%
CTE ~ Season + TUT + TIS + DSwamp + DInd	361,1	3,3	0,0%
CTE ~ Sct	361,1	3,3	0,0%
CTE ~ Season + DEco + TUT + TIS + Sct + DGamba + DInd	361,2	3,4	0,0%

Red duikers

Model	*AIC*	*ΔAIC*	*AIC weights*
PA ~ DEco + TLCT + TUT + TIS + DFWater + DOcean + DNPl + DInd	90,4	0,0	24,1%
PA ~ DEco + TLCT + TUT + DFWater + DOcean + DNPl + DInd	90,8	0,4	3,3%
PA ~ DEco + TLCT + TUT + DOcean + DNPl + DInd	90,9	0,5	2,0%
PA ~ DEco + TLCT + TUT + TIS + DOcean + DNPl + DInd	91,4	1,0	0,2%
PA ~ TVi + DEco + TLCT + TUT + TIS + DFWater + DOcean + DNPl + DInd	91,5	1,1	0,1%
PA ~ TVi + DEco + TLCT + TUT + DFWater + DOcean + DNPl + DInd	91,5	1,1	0,1%
PA ~ TLCT + TUT + DOcean + DNPl + DInd	91,6	1,2	0,1%
PA ~ DEco + TLCT + TUT + TCS + TIS + DFWater + DOcean + DNPl + DInd	91,8	1,4	0,0%
PA ~ TVi + DEco + TLCT + TUT + TCS + DFWater + DOcean + DNPl + DInd	92,3	1,9	0,0%
PA ~ TLCT + TUT + DFWater + DOcean + DNPl + DInd	92,4	2,0	0,0%
PA ~ TVi + DEco + TLCT + TUT + TIS + DOcean + DNPl + DInd	92,7	2,3	0,0%
PA ~ TVi + DEco + TLCT + TUT + TCS + TIS + DFWater + DOcean + DNPl + DInd	92,8	2,4	0,0%
PA ~ TVi + DEco + TLCT + TUT + TIS + DFWater + DOcean + DNPl + DGamba + DInd	92,9	2,5	0,0%
PA ~ DEco + TLCT + TUT + TCS + TIS + DFWater + DOcean + DNPl + DGamba + DInd	93,3	2,9	0,0%
PA ~ TVi + DEco + TLCT + TUT + TCS + DFWater + DOcean + DNPl + DGamba + DInd	93,5	3,1	0,0%
PA ~ TLCT + TUT + TIS + DFWater + DOcean + DNPl + DInd	93,5	3,1	0,0%
PA ~ TVi + DEco + TLCT + TUT + TCS + TIS + DOcean + DNPl + DInd	93,7	3,3	0,0%
PA ~ TVi + TLCT + TUT + TIS + DFWater + DOcean + DNPl + DInd	94,0	3,6	0,0%
PA ~ TVi + DEco + TLCT + TUT + TCS + TIS + DFWater + DOcean + DNPl + DGamba + DInd	94,2	3,8	0,0%
PA ~ TVi + DEco + TLCT + TUT + TCS + TIS + DFWater + DOcean + DSwamp + DNPl + DInd	94,4	4,0	0,0%

Red river hog

Model	*AIC*	*ΔAIC*	*AIC weights*
CST ~ TUT + DFWater + DSwamp + Sct + DNPl + DNStl + Dind + KAIIA + DNPk	436,4	0,0	24,1%
CST ~ TUT + DFWater + DSwamp + Sct + DNPl + DNStl + DInd + DNPk	437,3	0,9	0,3%
CST ~ TUT + TIS + DFWater + DSwamp + Sct + DNPl + DNStl + Dind + KAIIA + DNPk	437,8	1,4	0,0%
CST ~ TUT + TCS + DFWater + DSwamp + Sct + DNPl + DNStl + DInd + KAIIA + DNPk	438,0	1,6	0,0%
CST ~ TUT + TIS + DFWater + DSwamp + Sct + DNPl + DNStl + DInd + DNPk	438,8	2,4	0,0%
CST ~ TUT + TCS + TIS + DFWater + DSwamp + Sct + DNPl + DNStl + DInd + KAIIA + DNPk	439,2	2,8	0,0%
CST ~ TUT + TIS + DFWater + DSwamp + Sct + DNPl + KAIPFA + DNStl + DInd + KAIIA + DNPk	439,6	3,2	0,0%
CST ~ TUT + DFWater + DSwamp + Sct + DNPl + DNStl + DInd + KAIIA	439,9	3,5	0,0%
CST ~ TUT + DFWater + DSwamp + Sct + DNPl + DInd + DNPk	440,1	3,7	0,0%
CST ~ TUT + TCS + TIS + DFWater + DSwamp + Sct + DNPl + DNStl + DInd + DNPk	440,2	3,8	0,0%
CST ~ TUT + TIS + DFWater + DSwamp + Sct + DNPl + DInd + KAIIA + DNPk	440,6	4,2	0,0%
CST ~ TUT + DFWater + DSwamp + Sct + DNPl + DInd	440,8	4,4	0,0%
CST ~ TUT + TIS + DFWater + DSwamp + Sct + DNPl + DNStl + DInd + KAIIA	440,9	4,5	0,0%
CST ~ TUT + DFWater + DSwamp + Sct + DNPl + DNStl + KAIIA + DNPk	440,9	4,5	0,0%
CST ~ TUT + TCS + TIS + DFWater + DSwamp + Sct + DNPl + KAIPFA + DNStl + DInd + KAIIA + DNPk	441,0	4,6	0,0%
CST ~ TUT + DFWater + DSwamp + Sct + DNPl + DNPk	441,0	4,6	0,0%
CST ~ DEco + TUT + TCS + TIS + DFWater + DSwamp + Sct + DNPl + DNStl + DInd + KAIIA + DNPk	441,2	4,8	0,0%
CST ~ TUT + DFWater + DSwamp + Sct + DNPl + DNStl + DInd	441,2	4,8	0,0%
CST ~ DEco + TUT + TIS + DFWater + DSwamp + Sct + DNPl + KAIPFA + DNStl + DInd + KAIIA + DNPk	441,6	5,2	0,0%
CST ~ DEco + TUT + TCS + DFWater + DSwamp + Sct + DNPl + KAIPFA + DNStl + DInd + KAIIA + DNPk	441,8	5,4	0,0%

Water chevrotain

Model	*AIC*	*ΔAIC*	*AIC weights*
PA ~ LEct2 + TIS + DFWater	106,7	0,0	24,1%
PA ~ TIS	106,7	0,0	24,1%
PA ~ TIS + DNPl	106,7	0,0	24,1%
PA ~ TIS + DFWater	106,9	0,2	8,9%
PA ~ TIS + DFWater + DGamba	107,8	1,1	0,1%
PA ~ LEct2 + TIS + DFWater + DGamba	107,9	1,2	0,1%
PA ~ LEct2 + TIS	108,2	1,5	0,0%
PA ~ LEct2 + TIS + DFWater + DNPl	108,6	1,9	0,0%
PA ~ TIS + DGamba	108,7	2,0	0,0%
PA ~ LEct2 + TIS + DFWater + DNPl + DGamba	108,7	2,0	0,0%
PA ~ LEct2 + TIS + DFWater + DNPl + DNStl + DGamba	109,7	3,0	0,0%
PA ~ LEct2 + TIS + DFWater + DNStl + DGamba	109,9	3,2	0,0%
PA ~ LEct2 + TIS + DFWater + DSwamp + DNPl + DGamba	110,2	3,5	0,0%
PA ~ LEct2 + TIS + DGamba	110,2	3,5	0,0%
PA ~ LEct2 + TIS + DFWater + DNPl + DNStl	110,5	3,8	0,0%
PA ~ TIS + DFWater + DSwamp + DNPl + DNStl + DGamba	110,6	3,9	0,0%
PA ~ TIS + DFWater + DNPl + DNStl + DGamba	110,7	4,0	0,0%
PA ~ LEct2 + TIS + DFWater + DSwamp + DNPl + DNStl + DGamba	110,8	4,1	0,0%
PA ~ LEct2 + TIS + DFWater + DOcean + DSwamp + DNPl + DGamba	111,3	4,6	0,0%
PA ~ LEct2 + TIS + DFWater + DOcean + DNPl + DNStl + DGamba	111,5	4,8	0,0%

Side-striped jackal

Model	*AIC*	*ΔAIC*	*AIC weights*
PA ~ TVi + LEct2 + TIS + Sct + KAIPFA + DInd	79,5	0,0	24,1%
PA ~ TVi + LEct2 + TIS + Sct + KAIPFA + DInd + KAIIA	79,6	0,1	14,6%
PA ~ TVi + LEct2 + Sct + KAIPFA	79,8	0,3	5,4%
PA ~ TVi + LEct2 + TIS + Sct + KAIPFA	80,0	0,5	2,0%
PA ~ TVi + LEct2 + TIS + DFWater + Sct + KAIPFA + DInd + KAIIA	80,4	0,9	0,3%
PA ~ TVi + LEct2 + TIS + DFWater + Sct + KAIPFA + DInd	80,5	1,0	0,2%
PA ~ TVi + LEct2 + Sct + KAIPFA + DInd	80,5	1,0	0,2%
PA ~ TVi + LEct2 + TIS + Sct + KAIPFA + KAIIA	80,7	1,2	0,1%
PA ~ TVi + LEct2 + TIS + KAIPFA + DInd + KAIIA	81,0	1,5	0,0%
PA ~ TVi + LEct2 + TIS + DFWater + Sct + DNPl + KAIPFA + DInd	81,1	1,6	0,0%
PA ~ TVi + LEct2 + TIS + KAIPFA + DInd	81,3	1,8	0,0%
PA ~ TVi + LEct2 + Sct + KAIPFA + DInd + KAIIA	81,4	1,9	0,0%
PA ~ TVi + LEct2 + TIS + Sct + DNPl + KAIPFA + DInd + KAIIA	81,5	2,0	0,0%
PA ~ TVi + LEct2 + TIS + DFWater + Sct + KAIPFA + DGamba + DInd + KAIIA	81,7	2,2	0,0%
PA ~ TVi + LEct2 + TIS + DFWater + Sct + DNPl + KAIPFA + DInd + KAIIA	81,7	2,2	0,0%
PA ~ TVi + LEct2 + TIS + DFWater + Sct + DNPl + KAIPFA + DGamba + DInd	81,9	2,4	0,0%
PA ~ TVi + LEct2 + DFWater + Sct + KAIPFA + DInd + KAIIA	82,0	2,5	0,0%
PA ~ TVi + LEct2 + TIS + DFWater + Sct + KAIPFA + KAIIA	82,0	2,5	0,0%
PA ~ TVi + LEct2 + TIS + DFWater + Sct + DNPl + KAIPFA + DGamba + DInd + KAIIA	82,3	2,8	0,0%
PA ~ TVi + LEct2 + TIS + DFWater + KAIPFA + DInd + KAIIA	82,5	3,0	0,0%

Small terrestrial carnivores

Model	*AIC*	*ΔAIC*	*AIC weights*
CST ~ TVi + LEct2 + LEct5 + TIS + TTP + DFWater + DSwamp + KAIPFA + DInd + DNPk	231,1	0,0	24,1%
CST ~ TVi + LEct2 + LEct5 + TLCT + TIS + TTP + DFWater + KAIPFA + DInd + DNPk	231,1	0,0	24,1%
CST ~ TVi + LEct2 + TLCT + TIS + TTP + DFWater + KAIPFA + DInd + DNPk	231,1	0,0	24,1%
CST ~ TVi + LEct2 + LEct5 + TLCT + TIS + TTP + DFWater + DSwamp + KAIPFA + DInd + DNPk	231,4	0,3	5,4%
CST ~ TVi + LEct2 + LEct5 + TLCT + TIS + TTP + DFWater + DInd + DNPk	231,8	0,7	0,7%
CST ~ TVi + LEct2 + LEct5 + TLCT + TTP + DFWater + KAIPFA + DInd + DNPk	232,3	1,2	0,1%
CST ~ TVi + LEct2 + LEct5 + TIS + TTP + DFWater + DSwamp + KAIPFA + DNStl + DInd + DNPk	232,4	1,3	0,0%
CST ~ TVi + LEct2 + LEct5 + TLCT + TIS + TTP + DFWater + DSwamp + DInd + DNPk	232,5	1,4	0,0%
CST ~ TVi + LEct5 + TLCT + TIS + TTP + DFWater + DSwamp + KAIPFA + DNStl + DInd + DNPk	232,6	1,5	0,0%
CST ~ TVi + LEct2 + TLCT + TIS + TTP + DFWater + Dswamp + KAIPFA + DInd + DNPk	232,6	1,5	0,0%
CST ~ TVi + LEct2 + LEct5 + TIS + TTP + DFWater + DSwamp + Sct + KAIPFA + DNStl + DInd + DNPk	232,7	1,6	0,0%
CST ~ TVi + LEct2 + LEct5 + TLCT + TIS + TTP + DFWater + DSwamp + KAIPFA + DNStl + DInd + DNPk	232,7	1,6	0,0%
CST ~ TVi + LEct2 + LEct5 + TLCT + TIS + TTP + DFWater + DSwamp + KAIPFA + DInd	232,7	1,6	0,0%
CST ~ TVi + LEct2 + LEct5 + TLCT + TIS + TTP + DFWater + DSwamp + Sct + KAIPFA + DInd + DNPk	232,8	1,7	0,0%
CST ~ TVi + LEct2 + LEct5 + TLCT + TTP + DFWater + DSwamp + KAIPFA + DInd + DNPk	232,8	1,7	0,0%
CST ~ TVi + TLCT + TIS + TTP + DFWater + DInd	232,9	1,8	0,0%
CST ~ TVi + LEct5 + TLCT + TIS + TTP + DFWater + DSwamp + Sct + KAIPFA + DNStl + DInd + DNPk	233,1	2,0	0,0%
CST ~ TVi + LEct5 + LEct2 + TLCT + TIS + TTP + DFWater + KAIPFA + DNStl + DInd + DNPk	233,1	2,0	0,0%
CST ~ TVi + TLCT + TIS + TTP + DFWater + DInd + DNPk	233,2	2,1	0,0%
CST ~ TVi + LEct2 + TLCT + TIS + TTP + DFWater + DInd + DNPk	233,4	2,3	0,0%

Gorilla

Model	*AIC*	*ΔAIC*	*AIC weights*
PA ~ TLCT + TTP + DInd + DNPk	92,4	0,0	24,1%
PA ~ TLCT + TTP + DGamba + DInd + DNPk	92,5	0,1	14,6%
PA ~ TLCT + TTP	92,6	0,2	8,9%
PA ~ TLCT + TTP + DNStl + DInd + DNPk	92,9	0,5	2,0%
PA ~ TLCT + TTP + DNPk	92,9	0,5	2,0%
PA ~ TLCT + TTP + DNStl + DGamba + DInd + DNPk	93,1	0,7	0,7%
PA ~ TLCT + TTP + DNStl + DGamba + DNPk	93,3	0,9	0,3%
PA ~ TLCT + TTP + DGamba + DNPk	93,8	1,4	0,0%
PA ~ TLCT + TTP + Sct + DNStl + DInd + DNPk	93,9	1,5	0,0%
PA ~ TLCT + TTP + Sct + DGamba + DInd + DNPk	94,4	2,0	0,0%
PA ~ TLCT + TTP + Sct + DNStl + DGamba + DInd + DNPk	94,5	2,1	0,0%
PA ~ TLCT + TTP + DInd	94,5	2,1	0,0%
PA ~ TLCT + TTP + Sct + DNStl + DGamba + DNPk	94,7	2,3	0,0%
PA ~ DEco + TLCT + TTP + DNStl + DGamba + DInd + DNPk	95,0	2,6	0,0%
PA ~ TLCT + DGamba + DInd + DNPk	95,0	2,6	0,0%
PA ~ TLCT + DInd + DNPk	95,0	2,6	0,0%
PA ~ DEco + TLCT + TTP + Sct + DNStl + DInd + DNPk	95,1	2,7	0,0%
PA ~ TLCT + TTP + DNStl + DGamba + DInd	95,5	3,1	0,0%
PA ~ DEco + TLCT + TTP + Sct + DNStl + DGamba + DInd + DNPk	95,9	3,5	0,0%
PA ~ DEco + TLCT + TTP + DFWater + Sct + DNStl + DInd + DNPk	96,1	3,7	0,0%

Chimpanzee

Model	*AIC*	*ΔAIC*	*AIC weights*
PA ~ DEco + LEct5 + TUT + TTP + DSwamp	105,9	0,0	24,1%
PA ~ DEco + LEct5 + TUT + TIS + TTP + Dswamp	106,1	0,2	8,9%
PA ~ LEct5 + DNStl + KAIPFA	106,2	0,3	5,4%
PA ~ DEco + LEct5	106,3	0,4	3,3%
PA ~ DEco + LEct5 + TUT + TIS + TTP + DSwamp + DGamba + DInd	106,8	0,9	0,3%
PA ~ DEco + LEct5 + TTP + DSwamp	106,8	0,9	0,3%
PA ~ DEco + LEct5 + TUT + TIS + TTP	106,9	1,0	0,2%
PA ~ DEco + LEct5 + TUT + DSwamp	107,0	1,1	0,1%
PA ~ LEct5	107,0	1,1	0,1%
PA ~ DEco + LEct5 + TUT + TTP	107,1	1,2	0,1%
PA ~ DEco + LEct5 + TUT + TIS + TTP + DSwamp + DGamba	107,3	1,4	0,0%
PA ~ DEco + LEct5 + TTP	107,3	1,4	0,0%
PA ~ DEco + LEct5 + TUT + TTP + DSwamp + DGamba + DInd	107,4	1,5	0,0%
PA ~ DEco + LEct5 + TUT + TTP + DSwamp + DGamba	107,4	1,5	0,0%
PA ~ DEco + LEct5 + TUT + TIS + TTP + DGamba	107,4	1,5	0,0%
PA ~ DEco + LEct5 + DSwamp	107,5	1,6	0,0%
PA ~ DEco + LEct5 + TIS + TTP + DSwamp	107,7	1,8	0,0%
PA ~ DEco + LEct5 + TUT + TIS + TTP + DSwamp + DNStl + DGamba + DInd	107,8	1,9	0,0%
PA ~ DEco + LEct5 + TUT + TIS + DSwamp	107,9	2,0	0,0%
PA ~ LEct5 + TUT + TTP + DSwamp	107,9	2,0	0,0%

Collared mangabey

Model	*AIC*	*ΔAIC*	*AIC weights*
PA ~ TVi + DEco + TTP + DOcean + DNPl + DNStl	217,8	0,0	24,1%
PA ~ TVi + DEco + TCS + TTP + DOcean + DNPl + DNStl	217,9	0,1	14,6%
PA ~ TVi + DEco + TUT + TTP + DOcean + DNPl + DNStl	218,0	0,2	8,9%
PA ~ TVi + DEco + TTP + DOcean + DNPl + DNStl	218,2	0,4	3,3%
PA ~ DEco + TTP + DOcean + DNPl + DNStl	218,3	0,5	2,0%
PA ~ DEco + TCS + TTP + DOcean + DNPl + DNStl	218,3	0,5	2,0%
PA ~ TVi + DEco + TUT + TCS + TTP + DOcean + DNPl + DNStl	218,4	0,6	1,2%
PA ~ TVi + DEco + TCS + TTP + DOcean + DNPl + DNStl	218,5	0,7	0,7%
PA ~ TVi + DEco + TCS + TTP + DOcean + DNPl + KAIPFA + DNStl	218,6	0,8	0,4%
PA ~ TVi + DEco + TUT + TTP + DOcean + DNPl + KAIPFA + DNStl	218,6	0,8	0,4%
PA ~ TVi + DEco + TUT + TCS + TTP + DOcean + DNPl + KAIPFA + DNStl	218,8	1,0	0,2%
PA ~ TVi + DEco + TUT + TCS + TTP + DNPl + DNStl	219,5	1,7	0,0%
PA ~ TVi + TTP + DOcean + DNPl + DNStl	219,5	1,7	0,0%
PA ~ TVi + DEco + TUT + TIS + TTP + DOcean + DNPl + KAIPFA + DNStl	219,6	1,8	0,0%
PA ~ TVi + DEco + TUT + TCS + TIS + TTP + DOcean + DNPl + DNStl	219,6	1,8	0,0%
PA ~ TVi + DEco + TUT + TCS + TIS + TTP + DOcean + DNPl + KAIPFA + DNStl	220,1	2,3	0,0%
PA ~ TVi + DEco + TCS + TIS + TTP + DOcean + DNPl + KAIPFA + DNStl	220,1	2,3	0,0%
PA ~ TVi + DEco + TUT + TCS + TTP + DNPl + KAIPFA + DNStl	220,2	2,4	0,0%
PA ~ TVi + Season+ DEco + TUT + TCS + TTP + DOcean + DNPl + KAIPFA + DNStl	220,6	2,8	0,0%
PA ~ TVi + TTP + DOcean + DNPl	220,6	2,8	0,0%

Spot-nosed monkey

Model	*AIC*	*ΔAIC*	*AIC weights*
CST ~ TVi + DEco + DOcean + DSwamp + Sct	149,1	0,0	24,1%
CST ~ TVi + DEco + LEct5 + DOcean + DSwamp + Sct	150,0	0,9	0,3%
CST ~ TVi + DEco + TTP + DOcean + DSwamp + Sct	150,0	0,9	0,3%
CST ~ DEco + DOcean + DSwamp + Sct	150,7	1,6	0,0%
CST ~ TVi + DEco + LEct5 + TTP + DOcean + DSwamp + Sct	150,7	1,6	0,0%
CST ~ TVi + DEco + DSwamp + Sct	150,7	1,6	0,0%
CST ~ TVi + DEco + LEct5 + TTP + DOcean + DSwamp	150,8	1,7	0,0%
CST ~ TVi + DOcean + DSwamp + Sct	150,9	1,8	0,0%
CST ~ Sct	151,0	1,9	0,0%
CST ~ TVi + DEco + TTP + DSwamp + Sct	151,1	2,0	0,0%
CST ~ TVi + DEco + TCS + TTP + DOcean + DSwamp + Sct	151,3	2,2	0,0%
CST ~ TVi + DEco + DOcean + DSwamp	151,3	2,2	0,0%
CST ~ TVi + DEco + DOcean + Sct	151,4	2,3	0,0%
CST ~ TVi + DEco + LEct5 + TCS + DOcean + DSwamp + Sct	151,5	2,4	0,0%
CST ~ DEco + TTP + DOcean + DSwamp + Sct	151,5	2,4	0,0%
CST ~ TVi + DEco + LEct5 + TCS + TTP + DOcean + DSwamp	151,6	2,5	0,0%
CST ~ TVi + DEco + TTP + DOcean + Dswamp	151,9	2,8	0,0%
CST ~ TVi + DEco + LEct5 + TCS + TTP + DOcean + DSwamp + Sct	152,0	2,9	0,0%
CST ~ TVi + DEco + LEct5 + TIS + TTP + DOcean + DSwamp + Sct	152,0	2,9	0,0%
CST ~ TVi + TTP + DOcean + DSwamp + Sct	152,2	3,1	0,0%

All monkeys

Model	*AIC*	*ΔAIC*	*AIC weights*
CST ~ TVi + Season + DEco + TLCT + DNStl	231,6	0,0	24,1%
CST ~ Season + DEco + TLCT + DNStl	231,7	0,1	14,6%
CST ~ TVi + Season + DEco + TLCT + DOcean + DNStl	232,0	0,4	3,3%
CST ~ TVi + Season + DEco + DOcean + DNStl	232,2	0,6	1,2%
CST ~ TVi + Season + DEco + TLCT + TUT + DOcean + DNStl	232,3	0,7	0,7%
CST ~ Season + DEco + TLCT	232,4	0,8	0,4%
CST ~ Season + DEco + TLCT + DInd	232,4	0,8	0,4%
CST ~ TVi + Season + DEco + TUT + DOcean + DNStl	232,5	0,9	0,3%
CST ~ TVi + Season + DEco + TLCT + TUT + DNStl	232,5	0,9	0,3%
CST ~ Season + DEco + TLCT + DOcean + DNStl	232,6	1,0	0,2%
CST ~ TVi + Season + DEco + TLCT	233,0	1,4	0,0%
CST ~ TVi + Season + DEco + TLCT + DOcean + DNStl + DInd	233,5	1,9	0,0%
CST ~ Season + DEco + TLCT + TUT + DOcean + DNStl	233,5	1,9	0,0%
CST ~ TVi + Season + DEco + TLCT + TUT + DOcean + DNStl + DInd + DNPk	234,3	2,7	0,0%
CST ~ TVi + Season + DEco + TLCT + TUT + TCS + DOcean + DNStl + DInd	234,3	2,7	0,0%
CST ~ TVi + Season + DEco + TLCT + TUT + DOcean + DNStl + DInd	234,3	2,7	0,0%
CST ~ TVi + Season + DEco + TLCT + TUT + DNStl + DInd	234,4	2,8	0,0%
CST ~ TVi + Season + DEco + TLCT + DOcean + DNStl + DInd + DNPk	234,5	2,9	0,0%
CST ~ TVi + Season + DEco + TUT + DOcean + DNStl + DInd	234,5	2,9	0,0%
CST ~ TVi + DEco + TLCT + DNStl	234,6	3,0	0,0%

Brush-tailed porcupine

Model	*AIC*	*ΔAIC*	*AIC weights*
PA ~ TVi + TLCT + TUT + TIS + TTP + DFWater + Sct + DNPl + KAIPFA	240,3	0,0	24,1%
PA ~ TLCT + TUT + TIS + TTP + DFWater + Sct + DNPl + KAIPFA	240,4	0,1	14,6%
PA ~ TLCT + TUT + TIS + TTP + Sct + DNPl + KAIPFA	240,8	0,5	2,0%
PA ~ TVi + TLCT + TUT + TIS + TTP + Sct + DNPl + KAIPFA	241,4	1,1	0,1%
PA ~ TVi + TUT + TIS + TTP + DFWater + Sct + DNPl + KAIPFA	241,7	1,4	0,0%
PA ~ TVi + TUT + TIS + TTP + Sct + DNPl + KAIPFA	242,1	1,8	0,0%
PA ~ TUT + TIS + TTP + Sct + DNPl + KAIPFA	242,3	2,0	0,0%
PA ~ TUT + TIS + TTP + Sct + DNPl + KAIPFA	242,6	2,3	0,0%
PA ~ TUT + TIS + TTP + DFWater + Sct + DNPl + KAIPFA	242,7	2,4	0,0%
PA ~ TIS + TTP + Sct + DNPl + KAIPFA	243,1	2,8	0,0%
PA ~ TLCT + TUT + TIS + TTP + Sct + KAIPFA	243,5	3,2	0,0%
PA ~ TIS + TTP + Sct + DNPl	244,2	3,9	0,0%
PA ~ TIS + TTP + Sct + KAIPFA	244,5	4,2	0,0%
PA ~ TIS + TTP + Sct	244,7	4,4	0,0%
PA ~ TLCT + TUT + TIS + TTP + DFWater + Sct + DNPl	244,9	4,6	0,0%
PA ~ TVi + TLCT + TUT + TIS + TTP + DFWater + Sct + DNPl + KAIPFA	245,0	4,7	0,0%
PA ~ TUT + TIS + TTP + Sct + KAIPFA	245,1	4,8	0,0%
PA ~ TLCT + TUT + TIS + TTP + DNPl + KAIPFA	245,2	4,9	0,0%
PA ~ TLCT + TUT + TIS + TTP + Sct + DNPl	245,3	5,0	0,0%
PA ~ TVi + TLCT + TUT + TIS + TTP + DFWater + DOcean + Sct + DNPl + KAIPFA	245,9	5,6	0,0%

Giant-pouched rat

Model	*AIC*	*ΔAIC*	*AIC weights*
PA ~ TVi + Season + LEct5 + TTP + Sct + DNPl + DInd	141,1	0,0	24,1%
PA ~ Season + LEct5 + TTP + Sct + DNPl + DInd	141,2	0,1	14,6%
PA ~ TVi + Season + LEct5 + TTP + Sct + DNPl + DNStl + DInd	143,8	2,7	0,0%
PA ~ TVi + Season + LEct5 + TCS + TTP + Sct + DNPl + DInd	144,4	3,3	0,0%
PA ~ Season + LEct5 + TTP + Sct + DNPl + DNStl + DInd	144,5	3,4	0,0%
PA ~ TVi + Season + DEco + LEct5 + TCS + TTP + Sct + DNPl + DNStl + DInd	144,7	3,6	0,0%
PA ~ TVi + LEct5 + TTP + Sct + DNPl + DInd	144,8	3,7	0,0%
PA ~ TVi + Season + LEct5 + TTP + DNPl + DInd	145,0	3,9	0,0%
PA ~ TVi + Season + LEct5 + TCS + TTP + Sct + DNPl + DNStl + DInd	145,2	4,1	0,0%
PA ~ TVi + Season + DEco + LEct5 + TTP + Sct + DNPl + DNStl + DInd	145,4	4,3	0,0%
PA ~ LEct5 + TTP + Sct + DNPl + DInd	145,5	4,4	0,0%
PA ~ TVi + Season + TTP + Sct + DNPl + DInd	145,6	4,5	0,0%
PA ~ TVi + Season + DEco + LEct5 + TCS + TIS + TTP + DNPl + DNStl + DInd	145,8	4,7	0,0%
PA ~ TVi + Season + DEco + LEct5 + TCS + TTP + Sct + DNPl + DInd	145,8	4,7	0,0%
PA ~ Season + TTP + Sct + DNPl + DInd	145,9	4,8	0,0%
PA ~ Season + LEct5 + TTP + DNPl + DInd	146,0	4,9	0,0%
PA ~ TVi + Season+ LEct5 + TCS + TIS + TTP + Sct + DNPl + DNStl + DInd	146,0	4,9	0,0%
PA ~ Season+ LEct5 + TCS + TTP + Sct + DNPl + DNStl + DInd	146,1	5,0	0,0%
PA ~ TVi + Season+ DEco + LEct5 + TIS + TTP + Sct + DNPl + DNStl + DInd	146,3	5,2	0,0%
PA ~ TVi + Season+ LEct5 + TTP + DNPl + DNStl + DInd	146,5	5,4	0,0%
